# Animal versus plant-based protein and risk of cardiovascular disease and type 2 diabetes: a systematic review of randomized controlled trials and prospective cohort studies

**DOI:** 10.29219/fnr.v67.9003

**Published:** 2023-03-28

**Authors:** Christel Lamberg-Allardt, Linnea Bärebring, Erik Kristoffer Arnesen, Bright I. Nwaru, Birna Thorisdottir, Alfons Ramel, Fredrik Söderlund, Jutta Dierkes, Agneta Åkesson

**Affiliations:** 1 Department of Food and Nutrition, University of Helsinki, Finland; 2 Department of Internal Medicine and Clinical Nutrition, Institute of Medicine, Sahlgrenska Academy, University of Gothenburg, Sweden; 3 Department of Nutrition, Institute of Basic Medical Sciences, University of Oslo, Norway; 4 Krefting Research Centre, Institute of Medicine, University of Gothenburg, Sweden; 5 Health Science Institute, University of Iceland, Iceland; 6 Faculty of Food Science and Nutrition, University of Iceland, Iceland; 7 Unit of Cardiovascular and Nutritional Epidemiology, Institute of Environmental Medicine, the Karolinska Institute, Sweden; 8 Centre for Nutrition, Department of Clinical Medicine, University of Bergen, Norway and Department of Laboratory Medicine and Pathology, Haukeland University Hospital, Norway

**Keywords:** dietary protein, plant protein, cardiovascular disease mortality, incidence of type 1 diabetes, blood lipids

## Abstract

**Objectives:**

To systematically review the evidence on the effect of replacing the intake of animal protein with plant protein on cardiovascular disease (CVD) and type 2 diabetes (T2D) and their intermediate risk factors.

**Methods:**

We searched MEDLINE, Embase, Cochrane Central Register of Controlled Trials, and Scopus up to 12th May 2022 for randomized controlled trials (RCTs) or prospective cohort studies that investigated replacement of animal protein with plant protein from foods. Outcomes were CVDs, T2D, and in RCTs also the effects on blood lipids, glycemic markers, and blood pressure. Risk of bias was evaluated with the Cochrane’s RoB2, ROBINS-I, and USDA’s RoB-NObS tools. Random-effects meta-analyses assessed the effects of plant vs. animal proteins on blood lipids in RCTs. The evidence was appraised according to the World Cancer Research Fund’s criteria.

**Results:**

After screening 15,090 titles/abstracts, full text of 124 papers was scrutinized in detail, from which 13 RCTs and seven cohort studies were included. Eight of the RCTs had either some concern or high risk of bias, while the corresponding evaluation of cohort studies resulted in moderate risk of bias for all seven. Meta-analyses of RCTs suggested a protective effect on total cholesterol (mean difference -0.11 mmol/L; 95% CI -0.22, -0.01) and low-density lipoprotein cholesterol (-0.14 mmol/L; 95% CI -0.25, -0.02) by replacing animal protein with plant protein. The substitution of animal protein with plant protein (percentage of energy intake) in cohort studies was associated with lower CVD mortality (*n* = 4) and lower T2D incidence (*n* = 2). The evidence was considered *limited-suggestive* for both outcomes.

**Conclusion:**

Evidence that the substitution of animal protein with plant protein reduces risk of both CVD mortality and T2D incidence is *limited-suggestive*. Replacing animal protein with plant protein for aspects of sustainability may also be a public health strategy to lower the risk of CVD mortality and T2D.

## Popular scientific summary

- This systematic review on animal vs. plant protein and cardiovascular disease (CVD), type-2 diabetes (T2D), and cardiometabolic risk factors comprised cohort studies with substitution models and interventions with replacement.
- The evidence linking substitution of animal with plant protein to lower CVD mortality and T2D incidence was deemed limited-suggestive.
- Replacement of animal protein with plant protein for sustainability may also be considered as a public health strategy to lower the risk of CVD and T2D.

The role of protein intake and its effect on health outcomes has been a long-standing research topic of interest and has been a high priority in nutrition research and disease prevention. In addition, efforts to combat climate change have identified protein intake as an important target, especially reducing protein of animal origin, since the production of animal protein generally is resource-intensive and environmentally impactful compared to plant protein sources (1). Compared to plant protein, animal protein sources are generally associated with larger carbon footprints, more land use, and larger blue water footprints (2).

Cardiovascular disease (CVD) and type 2 diabetes (T2D) are the major causes of morbidity and mortality worldwide and are associated with high societal costs (3). A recent systematic review (SR) and meta-analysis of observational studies indicated that habitual high intake of total and animal protein is associated with an increased risk of T2D (4). In contrast, Mousavi et al. (5) showed no association between dietary protein intake from different sources and risk of CVD in an SR of prospective studies. Likewise, in another recent SR, dietary protein intake from different sources showed no association with risk of coronary heart disease (CHD), but in subgroup analysis, there was a lower risk of CVD mortality with an increasing plant protein intake (6). The latter observation was further supported in an SR by Qi et al. (7) who demonstrated that higher plant protein intake was associated with a reduced risk of all-cause and CVD mortality. Equally, Chen et al. (8) presented evidence from prospective cohort studies that suggested that total protein intake was associated with an increased risk of all-cause mortality, mainly driven by an increased risk of CVD mortality by intake of animal protein. However, this SR showed that plant protein intake was inversely associated with all-cause and CVD mortality. The SR performed for the 2012 Nordic Nutrition Recommendations (NNR) on protein intake and several outcomes, including CVD, body weight, cancer, T2D, fractures, renal outcomes, physical training, muscular strength, and mortality concluded that many of the included studies found beneficial associations with plant protein intake (9).

In revising the NNR for the 2022 edition, the intake of animal protein vs. plant protein in adults was a prioritized subject by the NNR Committee for an SR. Criteria for shortlisting topics were published in 2021 (10). Briefly, it was deemed justified to perform a new SR if there were important new scientific data since NNR 2012 and no recent, relevant, and qualified SR available on the topic (11). A scoping review identified new data since 2011 that may be relevant. The aim of this SR was to examine the evidence for whether replacing animal protein with plant protein reduces the risk of CVD and T2D.

## Methods

The methodology for the present SR followed the guidelines developed for the NNR 2022 (12, 13) and the Preferred Reporting Items for SRs and Meta-Analyses (14, 15). A protocol was pre-registered online on PROSPERO (https://www.crd.york.ac.uk/prospero, CRD42021240630). A focused research question was developed by the NNR 2022 Committee, defining the population/participants, intervention/exposure, control, outcome, timeframe, study design, and setting (PI/ECOTSS), in an iterative process with the SR authors. The funding source for NNR 2022 was the Nordic Council of Ministers and governmental food and health authorities of Norway, Finland, Sweden, Denmark, and Iceland (10).

### Eligibility criteria

The inclusion and exclusion criteria are outlined in the PI/ECOTSS statement (Table 1). Briefly, prospective cohort studies and non-randomized and randomized controlled trials (RCTs) conducted in healthy adult populations (>18 years) were eligible for inclusion. Studies including subjects with mild hypercholesterolemia (as reported by the authors), who were not treated with cholesterol-lowering medication, were included in the analyses of RCTs. We excluded prospective cohort studies that did not report on substitution of animal protein with plant protein in relation to the outcomes, and those that were from settings otherwise not relevant for the Nordic/Baltic population. In this case, studies that evaluated a parallel comparison between the intake of animal and plant protein were excluded as no substitution was performed in such studies. For RCTs using soy protein as plant protein source, we included only RCTs intervening soy with zero or low isoflavone content and excluded those with moderate or high isoflavone content. For interventions using soy protein with different levels of isoflavones, only the group with the lowest isoflavone content was included to discount effects of isoflavones and focus on those of the protein (16). Outcomes included CVD (mortality and incidence), T2D, and related cardiometabolic risk factors.

**Table 1:** Eligibility criteria for population/participants, intervention/exposure, control, outcome, timeframe, study design, and settings

Plant vs animal protein						
Population	Intervention or exposure	Comparators	Outcomes	Timing	Setting	Study design
Adults, 18 years or older	Plant protein intake	Animal protein intake	Atherosclerotic CVD including: Major incident fatal and non-fatal CVD (combined or separate: myocardial infarction, stroke, coronary heart disease, and coronary artery bypass graft) CVD mortality Incident T2D Changes in insulin resistance, insulin sensitivity, HBA1c, fasting glucose, and insulin Changes in blood pressure and blood lipids	Intervention trials must have ≥4 weeks of follow-up and cohorts >12 months of follow-up	Relevant for the general population in the Nordic and Baltic countries	Randomized or non-randomized intervention trials For observational epidemiological studies, we will consider prospective cohort studies, nested case–control studies, and case–cohort studies

CVD, cardiovascular disease; T2D, type 2 diabetes; HbA1c, hemoglobin A1c.

### Information sources and search strategy

A comprehensive literature search of MEDLINE (Ovid), Embase (Ovid), Cochrane Central Register of Controlled Trials, and Scopus was performed by a research librarian from the Karolinska Institutet University Library up to the search date, initially on 26th–28th March 2021, updated on 12 May 2022. The search strategy (Supplementary file 1) was developed in collaboration with the authors, led by CL-A and LB, and was peer-reviewed by research librarians at the University of Oslo Library of Medicine and Science, Norway. There were no date or language limitations in the search strategy. Grey literature searches were not performed.

### Selection and data collection process

Two investigators (JB and BN) independently reviewed titles, abstracts, and full-text articles for inclusions according to the PI/ECOTSS statement (Table 1), first in a pilot test of 10% of the papers, using the web tool Rayyan (https://rayyan.qcri.org) in blinded mode. Potentially eligible papers were retrieved and read in full text by the same two reviewers. Disagreements about inclusion were resolved by a third reviewer (AÅ).

Another four authors (JD, EA, AR, and FS), in pairs, independently extracted data from the included studies into pre-specified Excel forms. Disagreements were solved by discussion. Among the variables extracted were study design, information on recruitment, dietary intake, interventions and controls, assessment of outcomes, follow-up, drop-out, confounders, etc.

### Study risk of bias assessment

Risk of bias in each included study was assessed by two authors (CLA and BT), working independently. The assessment tools used were Cochrane’s Risk of Bias 2.0 (17) and Risk of Bias In Non-randomized Studies of Interventions (18, 19) for intervention studies, while ‘Risk of Bias for Nutrition Observational Studies’ (RoB-NObS) (20) was used for prospective observational studies. The risk of bias in each individual study was classified as ‘low’, ‘some concerns’, or ‘high’. Risk of bias was visualized by using the web app Risk-of-bias VISualization (robvis) (21).

### Synthesis methods

We performed a qualitative synthesis of the included studies by describing the main characteristics. Following the recommendations of the Healthcare Research and Quality (AHRQ), the Cochrane Handbook, and the NNR 2022 Handbook, a meta-analysis was performed if >3 independent RCTs or >5 cohort studies were available (12, 22–24).

Consequently, quantitative syntheses were performed of RCTs reporting effects on total cholesterol, LDL-cholesterol, HDL-cholesterol, and triglycerides. Measures expressed in mg/dl were converted to mmol/l by dividing mg/dl by 38.67 for cholesterol and 88.57 mg/dl for triglycerides. We used the random-effects meta-analyses with variance (τ^2^) estimated by the restricted maximum likelihood method. For most parallel-group and crossover trials, we used pooled differences in means and standard deviations (SD) of follow-up values, while if post-intervention outcomes were not reported, we included change from baseline scores. The SDs were imputed from standard errors if not reported. Homogeneity was assessed by the Cochran Q test, and we used the *I*^2^ statistic to quantify variability explained by between-study heterogeneity. *I*^2^ of ≥50% was considered ‘substantial’, and ≥75% ‘considerable’. Potential small study bias was assessed by Egger’s test (significance level *P* > 0.1) and visual inspection of funnel plots.

For studies using soy with different amounts of isoflavones, we included only the intervention arm using the lowest isoflavone dose. Differences between plant protein sources were evaluated by subgroup analyses of soy vs. non-soy interventions, with between-group heterogeneity assessed by Cochran’s Q. The meta-analyses were performed with Stata/SE version 17.0 (StataCorp LLC, College Station, Texas, USA).

### Certainty assessment

We categorized the strength of evidence according to the World Cancer Research Fund’s grading: ‘*Convincing*’, ‘*Probable*’, ‘*Limited – suggestive*’, ‘*Limited – no conclusion*’, and ‘*Substantial effects unlikely*’ (9). The quality (risk of bias), quantity, consistency, and precision in the body of evidence were considered in categorizing the strength of evidence.

## Results

### Study selection search results

**Fig. 1:**
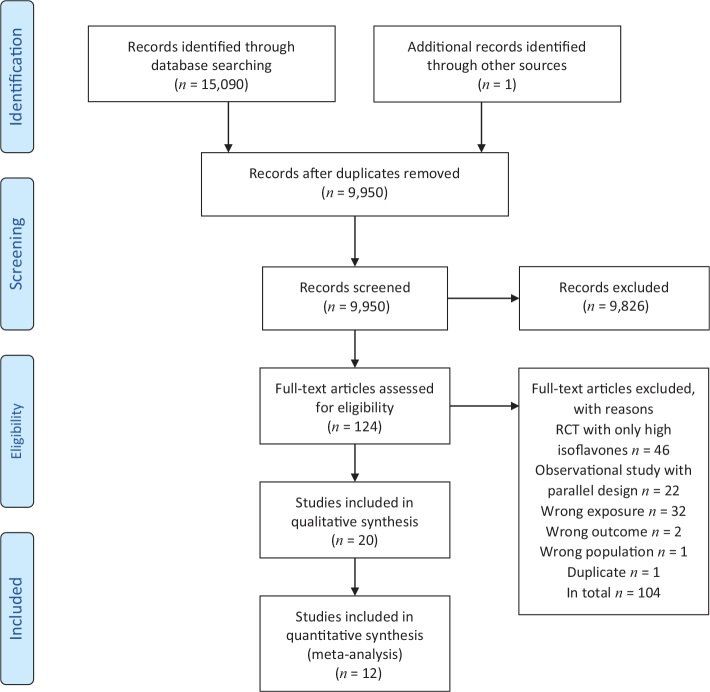
PRISMA flow diagram for database searches and study screening.

Figure 1 shows the literature search, screening, and the number of papers/studies excluded (including the reasons) as well as the studies retrieved and included in the SR. The potentially eligible studies excluded after the full-text assessment is listed together with reasons in the online supplement (Supplementary file 2).

### Study characteristics

In total, 20 publications were included (Tables 2 and 3). Out of these, 13 were RCTs (25–37), including between 23 and 140 subjects each (total, *n* = 906) (Table 2). Seven RCTs had a crossover design and six a parallel design. Seven of the RCTs were conducted in USA, three in Germany, two in Canada, and one in Brazil.

**Table 2:** Selected characteristics of the randomized controlled studies

Author Yearcountry	Population	Inclusion criteria	Design	Treatment / Exposures	Dietary assessment methods	Participants N	Age at inclusion/start of intervention	Follow-up time	Outcomes
Bähr et al. 2013 Germany	Hypercholesterolemic adults 18–80 years of age	Total cholesterol concentration of ≥5.2 mmol/L at screening	RCT, Cross-over	25 g protein/day, 5 g/100 mL lupin protein, or 5.1 g/100 mL milk protein	5-d weighted food record	66	Group AB: 49.7 (12.8) years Group BA: 49.4 (13.9) years	8 weeks	SBP, DBP at 8 weeks, TC, LDL-C, HDL-C, TG
Bähr et al. 2015 Germany	Hypercholesterolemic adults 18–80 years of age	Total cholesterol concentration of ≥5.2 mmol/L at screening	RCT, Cross-over	25 g/d lupin protein or milk protein or milk protein plus 1.6 g/d arginine	3-day food frequency protocol	72 (24/intervention period)	Group A: 54.0 (9.2) years Group B: 56.5 (13.2) years Group C: 59.8 (9.3) years	28 days + 6 weeks washout periods	SBP, DBP, TC, LDL-C, HDL-C, TG
Crouse et al. 1999 USA	Subjects with moderate hypercholesterolemia	Age 20–70 years with LDL cholesterol levels between 3.62 mmol/L (140 mg/dL) and 5.17 mmol/L (200 mg/dL) after following a run-in diet for 1 month (NCEP Step I low-fat, low-cholesterol diet consisting of 30% of energy as fat (polyunsaturated-monounsaturated-saturated fat ratio, 1:1:1) and 300 mg of cholesterol daily)	RCT, Parallell	25 g of soy isolate or 25 g casein per day Isolate soy protein containing either 3, 27, 37, or 62 mg isoflavones	Three 4-day records	28 (3 mg isoflavone); 31 (casein)	Mean (SD): 52 (11) years	9 weeks	TC, LDL-C, HDL-C, TG. Primary comparison was 62 mg isoflavones and casein.
Dent et al. 2001 USA	Perimenopausal women, normocholesterolemic, and mildly hypercholesterolemic	Experiencing ≥10 hot flushes and/or night sweats per wk, had irregular menses or cessation of menses for <1 y, had one or both ovaries remaining, had a body mass index (kg/m^2^) between 19 and 31, were willing to be randomly assigned to treatment, and were able to participate for 24 wk, follicle-stimulating hormone concentrations ≥30 iu/L.	RCT, Parallel	G1) Isoflavone-rich soy protein isolate G2) Isoflavone-poor soy protein isolate G3) Whey protein 40 g/day. Mean protein intake increased by 27 g/day.	5-day food records	24 (G2), 21 (G3)	50.2 ± 3.6 (41.9–61.6) years	24 weeks	TC, LDL-C, HDL-C, TG
Frota et al. 2015 Brazil	Mild or moderate hypercholesterolemic adults	Men aged 30–70 years or postmenopausal women age 45–70 years of age, mild to moderate hypercholesterolemia (LDL cholesterol ≥160 mg/dl, ≤190 mg/dl).	RCT, Cross-over	25 g protein in 2 servings of protein shakes daily (per cowpea shake: 12.6 protein, casein shake: 14.1 g protein)	24-h dietary recalls	38	57.0 (SEM 1.7) years	6 weeks	TC, LDL-C, HDL-C, TG, glucose (data not shown)
Gardner et al. 2001 USA	Postmenopausal, moderately hypercholesterolemic women	Postmenopausal (≥1 y since their last menstrual cycle), were aged <80 y, and had a body mass index (BMI; in kg/m^2^) of 20–31, LDL-cholesterol concentration of 3.37–4.92 mmol/L), and a triacylglycerol concentration <2.82 mmol/L	RCT, Parallell	42 g/day of soy protein isolate (2 × 21 g/d); Soy-: Isolated soy protein with trace amounts of isoflavone; Soy+: Isolated soy protein containing isoflavones Milk protein	3 day food records	30, 33	Milk: 57.7 (6.0) yrs, Soy-: 58.4 (7.2) yrs, Soy+: 62.6 (7.3) yrs	12 weeks	TC, LDL-C, HDL-C, TG
Gardner et al 2007 USA	Hypercholesterolemic adults	LDL-C concentration 4.14–5.69 mmol/L and Framingham risk score of ≤10% based on gender, age, LDL-C, HDL-C, blood pressure, and diabetes	RCT, Cross over	25 g protein from either milk type (32 oz whole bean soy drink, 28 oz soy protein isolate drink, 18.5 oz dairy milk). Isoflavones: 125 mg in whole bean soy milk, 39 mg in SPI drink)	Milk consumption logs and 3-day food records	28	52 (9) years	4 weeks	TC, LDL-C, HDL-C, TG, fasting insulin AUC, fasting glucose
Jenkins et al. 2010 Canada	Hypercholesterolemic adults	Men >21 y or postmenopausal women with LDL-C >3.5 mmol/L	RCT, Cross-over	30 g barley or casein protein per 2,000 kcal daily (18–19 g protein per 100 g)	7-day dietary history	23	56 (2) (range 41–69) years	6 weeks	TC, TG, LDL-C, HDL-C, SBP, DBP
Lichtenstein et al. 2002 USA	Moderately hypercholesterolemic Men and women	>50 years with LDL cholesterol levels greater than 3.36 mmol/L; and postmenopausal (for women)	RCT, Cross-over	Soy/-: Soy protein depleted of isoflavones, Soy/+: Soy protein enriched in isoflavones Animal/-: Animal protein without isoflavones, Animal/+: Animal protein enriched in isoflavones 2/3 of total protein intake (i.e., 10 E%, 25 g protein/4.2 MJ).	Not provided	42	62.7 (8.8) years	6 weeks	TC, LDL-C, HDL-C, TG
McVeigh et al. 2006 Canada	Healthy young males	Healthy males between the ages of 20 and 40 y and with a body mass index (BMI; in kg/m^2^) of 19–29.	RCT, Parallel	Low-iso SPI: Low isoflavone soy protein isolate; High-iso SPI: High isoflavone soy protein isolate According to body weight. High-iso: 0.75 mg isoflavones/kg/d. MPI: Milk protein isolate	3-d food record	70 (35; 35)	27.9 (5.7) years	57 days	TC, LDL-C, HDL-C, TG
Santo et al. 2008 USA	Healthy, young, sedentary males	Healthy, male, age 18–30 y, normocholesterolemic, BMI between 18 and 26 kg/m^2^	RCT, Parallel	Soy-: Isoflavone-poor soy protein isolate; Soy+: Isoflavone-rich soy protein isolate Milk: Milk protein isolate 25 g protein/day	3-day food records	30 (11; 10; 9)	Milk: 24.0 (0.9) years, Soy-: 23.6 (0.5) years, Soy+: 25.1 (0.8) years	28 days	TC, LDL-C, HDL-C, TG, Glucose
Steinberg et al 2003 USA	Healthy, postmenopausal women	Menopausal status, as defined by the absence of menstrual bleeding in the past 12 mo and follicle-stimulating hormone concentrations of ≥ 23 IU/L	RCT, Cross-over	Soy-: Isolated soy protein with trace amounts of isoflavones; Soy+: Isolated soy protein with naturally occurring isoflavones TMP: Total milk protein 25 g protein/day	3-day food records	28	54.9 (1.0) years	6 weeks	TC, LDL-C, HDL-C, TG
Weiße et al. 2010 Germany	Moderate, hypercholesterolemic adults	21 to 70 years of age with moderate hypercholesterolemia (5.7–7.9 mM)	RCT, Parallel	Lupin protein Casein protein 35 g protein/day	Diaries	43 (22; 21)	43.9 (11.8) years	6 weeks	TC, LDL-C, HDL-C, TG, Glucose

SBP, systolic blood pressure; DBP, diastolic blood pressure; TC, total cholesterol; LDL-C, low-density lipoprotein cholesterol; HDL-C, high-density lipoprotein cholesterol; TG, triacylglycerol; BMI, body mass index; RCT, randomized controlled trial; SD, standard deviation.

**Table 3:** Selected characteristics of the cohort studies

Author Yearcountry	Population	Design	Dietary assessment methods	Number recruited	Number analyzed	Age at inclusion/start of intervention	Follow-up time	Outcomes
Budhathoki et al. 2019 Japan	Eleven public health center areas across Japan, included in the Japan Public Health Center-based Prospective Cohort (JPHC) Study Residents aged 40 to 69 years	PC	138-item semiquantitative FFQ	140,420 (61,595 from cohort 1 and 78,825 from cohort 2)	70,696	Men: mean (SD): 55.6 (7.6) years, Women: mean (SD): 55.8 (7.7) years	Mean 18 years	CVD mortality
Huang et al. 2020 USA	National Institutes of Health-American Association of Retired Persons (NIH-AARP) Diet and Health Study Adults 50–71 years	PC	National Cancer Institute Diet History Questionnaire (DHQ) of 124 dietary items (FFQ)	566,398	416,104	Median (SD) ages: Men: 62.2≈(5.4) years, Women: 62.0 (5.4) years	16 years (median, 15.5 years; IQR, 15.5–15.8), 6,009,748 person years	CVD mortality
Malik et al. 2016 USA	Nurses’ Health Study (NHS), Nurses’ Health Study II (NHS II), and Health Professionals Follow-up Study (HPFS) Female registered nurses and male health professionals	PC	131-item FFQ	289,900 (NHS: 121,700, NHS II: 116,671, HPFS: 51,529)	205,802 (NHS: 72,992, NHS II: 92,088, HPFS: 40,722)	NHS: mean: ≈50.1 (30–55) years NHS II: mean: ≈36.0 (24–42) years HPFS: mean: ≈53.0 (40–75) years	4,146,216 person-years (18–24 years)	T2D incidence
Song et al. 2016 USA	Nurses’ Health Study (NHS) and Health Professionals Follow-up Study (HPFS) Female registered nurses and male health professionals	PC	131-item FFQ	173,229 (NHS: 121,700, HPFS: 51,529)	131,342 (NHS: 85,013, HPFS: 46,329)	NHS: 30–55 years, HPFS: 40–75 years	3,540,791 person-years	CVD mortality
Sun et al. 2021 USA	Women’s Health Initiative (WHI) Postmenopausal women	PC	122-item FFQ	137,481 (OS: 90,009; CT: 47,472)	102,521 (OS: 63,593; CT: 38,928)	50–79 years	1,876,205 person-years (18.1 years on average)	CVD mortality
Virtanen et al. 2017 Finland	Kuopio Ischaemic Heart Disease Risk Factor Study (KIHD) Middle-aged and older Finnish men	PC	4-d food record	2,682	2,332	42–60 years	Mean: 19.3 years	T2D incidence
Voortman et al 2021 The Netherlands	Participants from the Rotterdam Study, 3 different cohorts (RI, RII, RIII) Population based cohort study	PC	170-item semi-quantitative FFQ; RS-III, more comprehensive FFQ containing 389 items	14,926 complete dietary data from 9,701 participants	5,873	Mean age 61.6 (7.9) (60.8% females)	74,776 person-years	CHD incidence

FFQ, food frequency questionnaire; PC, prospective cohort; CHD, coronary heart disease; CVD, cardiovascular disease; T2D, type 2 diabetes.

There were seven reports (38–44) from seven cohort studies, including between 2,332 and 416,104 subjects (total, *n* = 720,663 for CVD mortality; *n* = 5,873 for CHD incidence; *n* = 281,341 for T2D incidence) with endpoint data (Table 3). The cohorts included subjects from USA, Japan, Finland, and the Netherlands.

### Types of intervention/exposures

Eight RCTs compared the effect of low-isoflavone soy protein supplementation to casein or milk protein supplementation on different outcomes (27, 28, 30, 31, 33–36) (Table 2). Three RCTs (25, 26, 37) compared the effect of lupin protein supplementation to milk protein or casein supplementation, and one (27) compared, in addition, the effect of lupin protein supplementation to milk protein + arginine supplementation on different outcomes. One RCT investigated the effect of barley protein supplementation in comparison to casein supplementation in bread (32), and one compared the effect of cowpea protein supplementation to casein supplementation (29) on different outcomes. The protein supplementation amount ranged between 25 and 30 mg/d for all studied protein sources. The outcomes in all studies were related to lipid metabolism. In some RCTs, the effects on glucose metabolism or blood pressure were studied.

Four reports from five prospective cohorts investigated the association between plant protein as E% substitution of animal protein and risk of CVD mortality (38, 39, 41, 42) (Table 3) and one on CHD incidence (44). Two reports from four prospective cohorts examined the association with plant protein intake as E% substitution of animal protein and the incidence of T2D (40, 43) (Table 3).

### Outcome assessment

The duration of the interventions in the RCTs ranged from 4 weeks to 24 weeks, all reporting on serum/plasma total cholesterol concentrations (total cholesterol), serum/plasma LDL (low-density lipoprotein)-cholesterol concentrations (LDL-cholesterol), serum/plasma HDL (high-density lipoprotein)-cholesterol concentrations (HDL-cholesterol), andserum/plasma triacylglycerol concentrations (triacylglycerol, TG). In addition, three studies (25, 26, 32) reported on effects on blood pressure, one on fasting serum/plasma insulin concentration (30), and four on blood glucose concentration (29, 30, 35, 37). If blood was drawn at several time points, only the results from the baseline and latest time point were considered. In the cohort studies, the follow-up time between assessment of diet and outcome ranged from 16 to 19.3 years (median or average in those where it was reported).

## Risk of bias in included studies

**Fig. 2:**
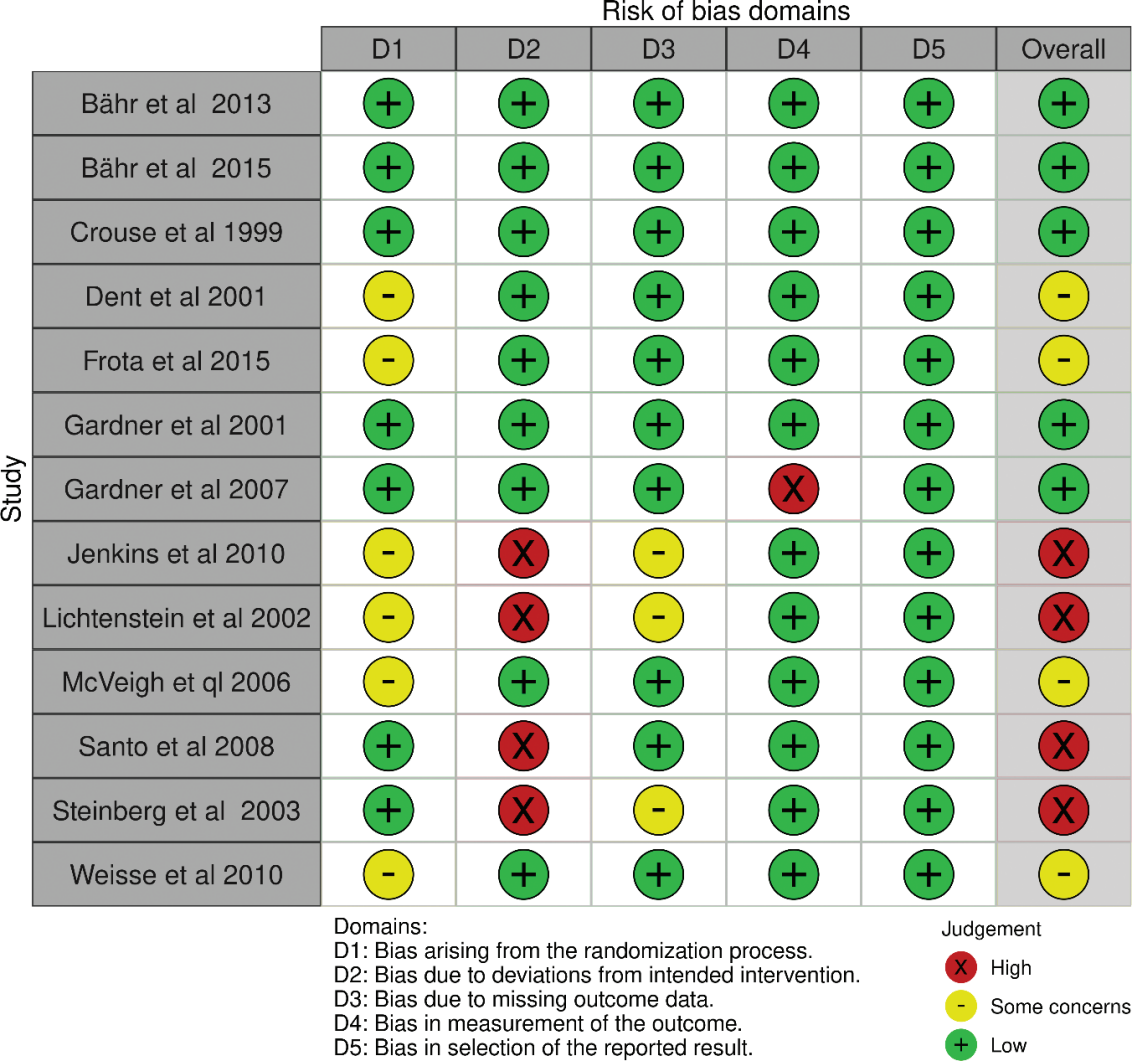
Risk of bias per domain and overall, for all included RCT studies. RCT, randomized controlled trials.

**Fig. 3:**
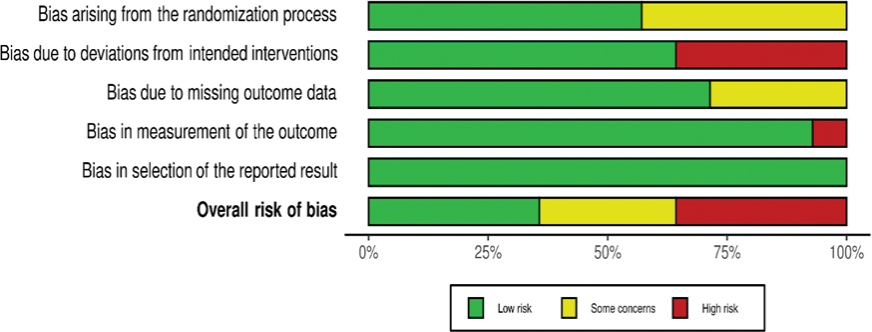
Summary of bias per domain and overall, for all included RCT studies. RCT, randomized controlled trials.

**Fig. 4:**
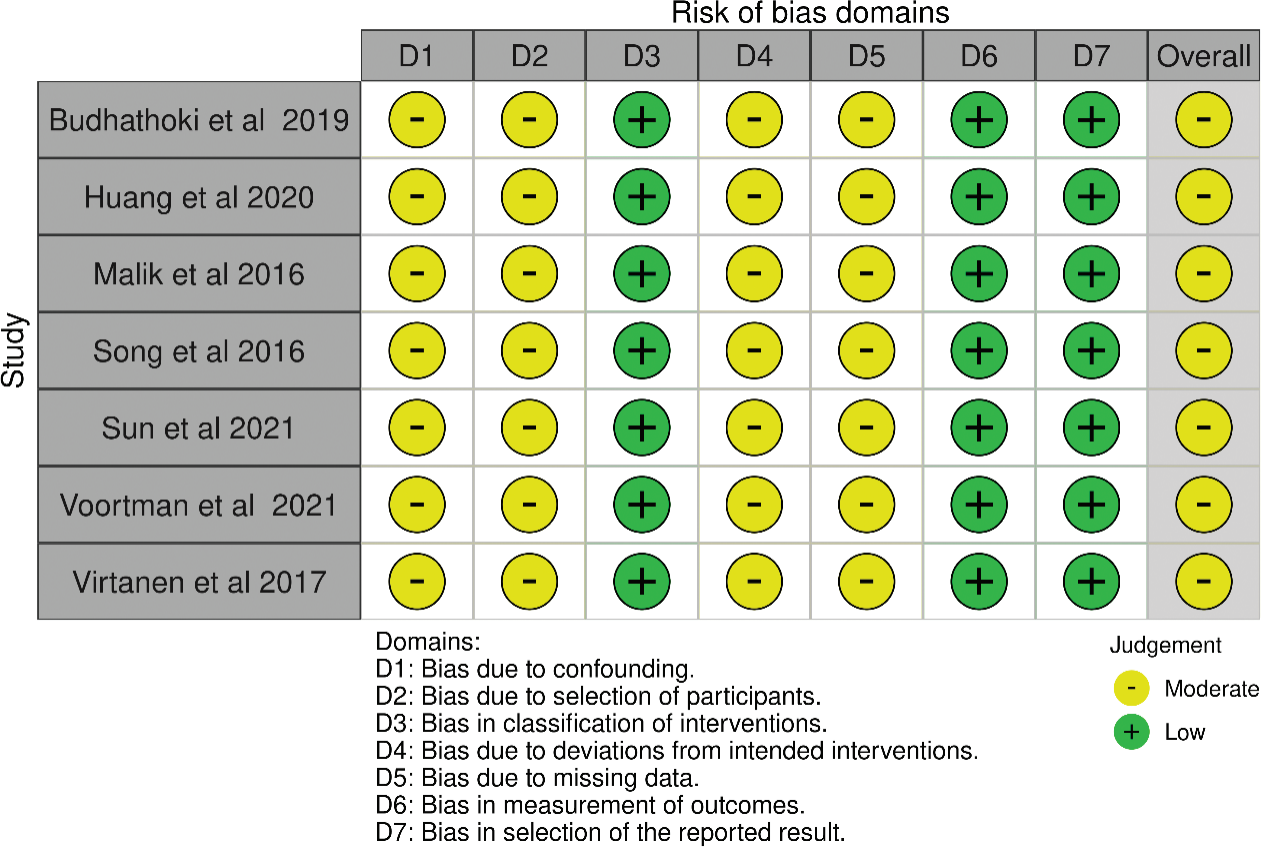
Risk of bias per domain and overall, for all included cohort studies.

**Fig. 5:**
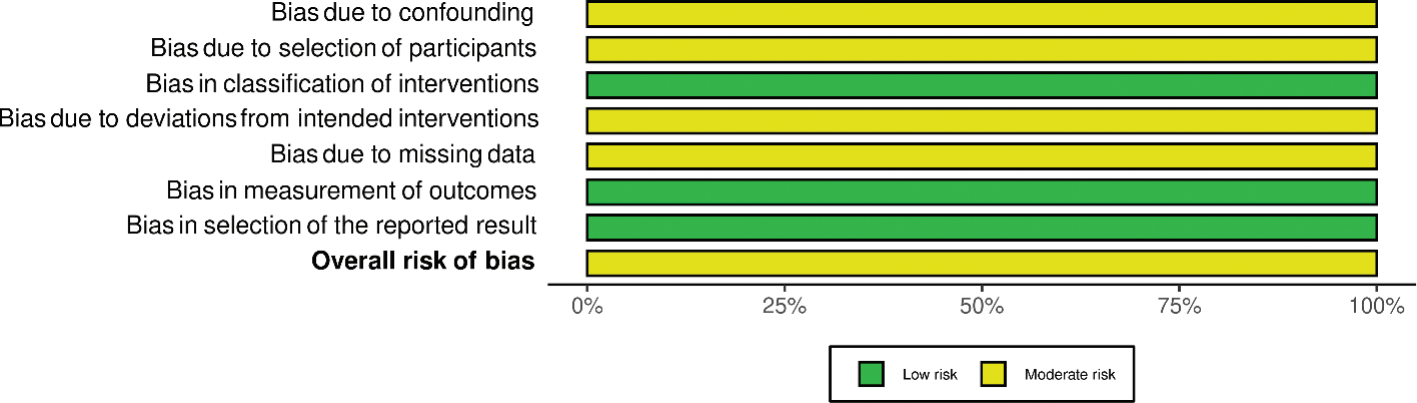
Summary risk of bias per domain and overall, for all included cohort studies.

The risk of bias assessment per domain in RCT studies is outlined in Figs. 2 and 3. Five RCTs had overall low concerns for risk of bias (25–27, 30, 31). Four RCTs had overall some concerns, due to the lack of information on the randomization process (28, 29, 34, 37). Four RCTs had overall high concern of bias, mostly due to non-adherence to the study intervention (32, 33, 35, 36). The risk of bias for all prospective cohort studies was moderate overall (Figs. 4 and 5).

### Plant proteins and blood lipids

The effect on total cholesterol, LDL-cholesterol, HDL-cholesterol, or triacylglycerol of soy protein in comparison to animal protein sources was studied in eight RCTs (27, 28, 30, 31, 33–36), of which three were cross-over studies (Tables 2 and 4). Three studies (25, 26, 37) explored the effect of lupin protein on blood lipids in hypercholesterolemic subjects, one studied the effect of barley protein (32), and one of cow-pea protein (29) (Tables 3 and 4).

**Table 4:** Summary of findings in randomized controlled trials

Author, year	Plant protein outcomes	Animal protein outcomes	Comparison between groups (*P*-value)	Summary of resultsa	Risk of bias
**Soy**					
Crouse et al. 1999	Soy, 3 mg isoflavones Mean (SD) at 9 weeks: TC: 6.10 (0.65) mmol/L LDL: 4.14 (0.57) mmol/L HDL: 1.19 (0.28) mmol/L TG: 1.72 (0.65) mmol/L	Casein Mean (SD) at 9 weeks: TC: 6.23 (0.70) mmol/L LDL: 4.27 (0.59) mmol/L HDL: 1.14 (0.23) mmol/L TG: 1.89 (0.84) mmol/l	TC: *P* = NS LDL: *P* = NS HDL: *P* = NS TG: *P* = NS	TC: ↔ LDL: ↔ HDL: ↔ TG: ↔	Low
Dent et al. 2001	SPI- = soy protein (low isoflavones) Estimated values from Fig. 1: Mean at 24 weeks: TC: 5.47 mmol/L LDL: 3.51 mmol/L Median: HDL: 1.07 mmol/L TG: 1.07 mmol/L	Whey protein Estimated values from Fig. 1: Mean at 24 weeks: TC: 5.46 mmol/L LDL: 3.52 mmol/L Median: HDL: 1.40 mmol/L TG: 1.35 mmol/L	TC: 0.96 LDL: 0.76 HDL: 0.99 TG: 0.9	TC: ↔ LDL: ↔ HDL: ↔ TG: ↔	Some
Gardner et al. 2001	Soy- Mean (SD) at 12 weeks: TC: 5.9 (0.9) mmol/L LDL: 3.8 (0.8) mmol/L HDL: 1.5 (0.2) mmol/L TG: 1.3 (0.6) mmol/L	Milk Mean (SD) at 12 weeks: TC: 5.9 (0.7) mmol/L LDL: 3.7 (0.6) mmol/L HDL: 1.5 (0.4) mmol/L TG: 1.4 (1.0) mmol/L	TC: n.s. between soy and milk LDL: n.s. between soy and milk HDL: 1.0 TG: 0.3	TC: ↔ LDL: ↔ HDL: ↔ TG: ↔	Low
Gardner et al. 2007	Mean (SD) at 4 weeks: LDL: Whole bean Soy milk: 4.17 (0.52) mmol/L Soy protein isolate milk: 4.17 (0.67) mmol/L Insulin AUC: Whole bean Soy milk: 44 (20) Soy protein isolate milk: 45 (25) Glucose. fasting: Whole beans milk: 5.2 (0.5) mmol/L Soy protein isolate milk: 5.1 (0.6) mmol/L	Dairy milk Mean (SD) at 4 weeks: LDL: 4.39 (0.62) mmol/L Insulin AUC: 44 (24) Glucose. fasting: 5.1 (0.6) mmol/L	Both soy milks vs. Dairy milk: LDL: *P* = 0.02 HDL: *P* = 0.8 TG: *P* = 0.4 Insulin: 0.9 Glucose: 0.4	LDL: ↓ HDL: ↔ TG: ↔ Insulin: ↔ Glucose: ↔	Low
Lichtenstein et al. 2002	Soy- Mean (SD) at 6 weeks: TC: 6.37 (1.12) mmol/L LDL: 4.34 (0.92) mmol/L HDL: 1.36 (0.34) mmol/L TG: 1.27 (0.50) mmol/L	Animal protein Mean (SD) at 6 weeks: TC: 6.47 (1.17) mmol/L LDL: 4.42 (0.97) mmol/L HDL: 1.33 (0.32) mmol/L TG: 1.44 (0.57) mmol/L	Between proteins: TC: *P* = 0.017. LDL: *P* = 0.042. HDL: *P* = 0.034. TG: *P* < 0.0001.	Between proteins: TC: ↓ LDL: ↓ HDL: TG: ↓	High
McVeigh et al. 2006	Low-iso Soy protein Least-squares mean (SE) at 57 days: TC: 4.47 (0.06) mmol/L LDL: 2.71 (0.05) mmol/L HDL: 1.15 (0.02) mmol/L TG: 1.35 (0.07) mmol/L	Milk protein Least-squares mean (SE) at 57 days: TC: 4.55 (0.06) mmol/L LDL: 2.86 (0.05) mmol/L HDL: 1.10 (0.02) mmol/L TG: 1.30 (0.07) mmol/L	TC: n.s. LDL: n.s. HDL: n.s. Non-HDL: n.s. TG: n.s.	TC: ↔ LDL: ↔ (↓ in equol excretors) HDL: ↔ Non-HDL: ↔ TG: ↔	Some
Santo et al. 2000	Low-isoflavone soy protein Mean (SEM) at 28 days: TC: 4.91 (0.34) mmol/L LDL: 2.92 (0.38) mmol/L HDL: 1.32 (0.11) mmol/L TG: 1.42 (0.27) mmol/L Glucose: 5.3 (0.2) mmol/L	Milk protein Mean (SEM) at 28 days: TC: 4.27 (0.25) mmol/L LDL: 2.66 (0.32) mmol/L HDL: 1.19 (0.15) mmol/L TG: 1.04 (0.18) mmol/L Glucose: 5.4 (0.3) mmol/L	Low-isoflavone soy vs. Milk: No differences	TC: ↔ LDL: ↔ HDL: ↔ TG: ↔ Glucose: ↔	High
Steinberg et al. 2003	Soy- Mean (SEM) at 6 weeks: TC: 4.92 (0.2) mmol/L LDL: 2.87 ± 0.1 mmol/L HDL: 1.55 ± 0.1 mmol/L TG: 1.08 ± 0.1 mmol/L Change from baseline: TC: 0.01 mmol/l LDL: -0.02 mmol/l	Milk protein Mean (SEM) at 6 weeks: TC: 5.00 ± 0.1 mmol/L LDL: 2.94 ± 0.1 mmol/L HDL: 1.61 ± 0.1 mmol/L TG: 0.98 ± 0.1 mmol/L Change from baseline TC: +0.08 mmol/l LDL: +0.04 mmol/l	All values non-significant between diets	TC: ↔ LDL: ↔ HDL: ↔ TG: ↔	High
Lupin					
Bähr et al. 2013	Lupin Change from baseline (mean (SD)) to 8 weeks: TC: 0.05 (0.44) mmol/L LDL: 0.08 (0.50) mmol/l HDL: -0.05 (0.19) mmol/L TG: 0.19 (0.45) mmol/L SBP/DBP: -8.4 (13.6)/ -2.7 (7.5) mmHg	Casein Change from baseline (mean (SD)) to 8 weeks: TC: 0.02 (0.49) mmol/L LDL: -0.06 (0.34) mmol/L HDL: -0.02 (0.13) mmol/L TG: 0.16 (0.77) mmol/L SBP/DBP: -5.9 (12.9)/ -1.5 (7.7) mmHg	TC: *P* = 0.52 LDL: *P* = 0.90 HDL: *P* = 0.20 TG: *P* = 0.77 SBP/DBP: *P* = 0.29/0.31	TC: ↔ LDL: ↔ HDL: ↔ (↑ at 4 weeks) TG: ↔ SBP: ↔ DBP: ↔	Low
Bähr et al. 2015	Lupin Mean (SD) at 4 weeks: TC: 6.13 (0.95) mmol/L LDL: 4.01 (0.87) mmol/L HDL: 1.35 (0.37) mmol/L TG: 1.69 (1.29) mmol/L SBP/DBP: 142.2 (20.8) / 87.0 (9.9) mmHg	Milk protein Mean (SD) at 4 weeks: TC: 6.23 (0.97) mmol/L LDL: 4.08 (0.95) mmol/L HDL: 1.36 (0.35) mmol/L TG: 1.77 (1.42) mmol/L SBP/DBP: 140.3 (19.2) / 86.8 (9.8) mm Hg	TC: *P* = 0.07 LDL: *P* = 0.044 HDL: *P* = 0.37 TG: *P* = 0.49 SBP/DBP: *P* = 0.35/0.84	TC: ↔ (*P* = 0.07) LDL: ↓ HDL: ↔ TG: ↔ SBP: ↔ DBP: ↔	Low
Weiße et al. 2010	Lupin protein Mean (SD) at 6 weeks: TC: 5.17 (0.59) mmol/L LDL: 3.30 (0.64) mmol/L HDL: 1.67 (0.42) mmol/L TG: 1.32 (0.72) mmol/L Glucose: 5.10 (0.75) mmol/L	Casein Mean (SD) at 6 weeks: TC: 5.32 (0.77) mmol/L LDL: 3.50 (0.73) mmol/L HDL: 1.54 (0.35) mmol/L TG: 1.26 (0.70) mmol/L Glucose: 5.14 (0.78) mmol/L	At 6 weeks TC: *P* = 0.509 LDL: *P* = 0.380 HDL: *P* = 0.294 TG, *P* = 0.715 Glucose: *P* = 0.861 Difference in change: TC: *P* = 0.9 LDL: *P* = 0.384 HDL: *P* = 0.150 TG: *P* = 0.068 Glucose: *P* = 0.992	Between groups, at 6 weeks TC: ↔ LDL: ↔ HDL: ↔ TG: ↔ Glucose: ↔ Difference in change: TC: ↔ LDL: ↔ HDL: ↔ TG: ↔ (*P* = 0.068)	Some
Cowpea					
Frota et al. 2015	Cowpea Mean (SEM) at 6 weeks: TC: 6.0 (0.11) mmol/L LDL: 3.67 (0.09) mmol/L HDL: 1.48 (0.04) mmol/L TG: 1.84 (0.16) mmol/L	Casein Mean (SEM) at 6 weeks: TC: 6.58 (0.12) mmol/L LDL: 4.26 (0.09) mmol/L HDL: 1.41 (0.04) mmol/L TG: 1.95 (0.25) mmol/L	Percentage changes TC: *P* < 0.001 LDL: *P* < 0.001 HDL: *P* = 0.044 TG: –	TC: ↓ LDL: ↓ HDL: ↑ TG: ↔	Some
Barley					
Jenkins et al. 2010	Barley Mean (SEM) at 4 weeks: TC: 5.9 (0.19) mmol/L LDL: 3.95 (0.16) mmol/L HDL: 1.30 (0.06) mmol/L TG: 1.42 (0.11) mmol/L Blood pressure SBP: 118 (2) mmHg DBP: 69 (2) mmHg	Casein Mean (SEM) at 4 weeks: TC: 5.79 (0.19) mmol/L LDL: 3.93 (0.18) mmol/L HDL: 1.27 (0.06) mmol/L TG: 1.32 (0.10) mmol/L Blood pressure SBP: 118 (3) mmHg DBP: 69 (2) mmHg	Difference between treatments TC: *P* = 0.57 LDL: *P* = 0.896 HDL: *P* = 0.184 TG: *P* = 0.334 Blood pressure SBP, *P* = 0.639 DBP, *P* = 0.418	TC: ↔ LDL: ↔ HDL: ↔ TG: ↔ SBP: ↔ DBP: ↔	High

SBP, systolic blood pressure; DBP, diastolic blood pressure; TC, total cholesterol; LDL-C, low-density lipoprotein cholesterol; HDL-C, high-density lipoprotein cholesterol; TG, triacylglycerol; AUC, area under curve; SE, standard error of mean; SD, standard deviation. ^a^Arrows indicate the direction of association.

**Fig. 6:**
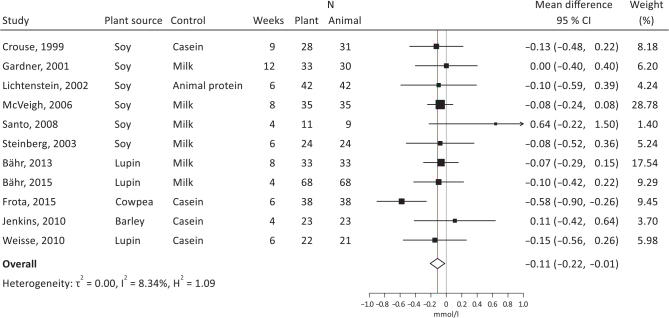
Meta-analysis of RCT studies of total cholesterol. Forest plot showing mean differences with 95% CI in total cholesterol (mmol/l) by replacing animal protein with plant protein. The summary effect estimate (white diamond) was estimated by a restricted maximum likelihood random-effects model. RCT, randomized controlled trials; CT, confidence interval.

**Fig. 7:**
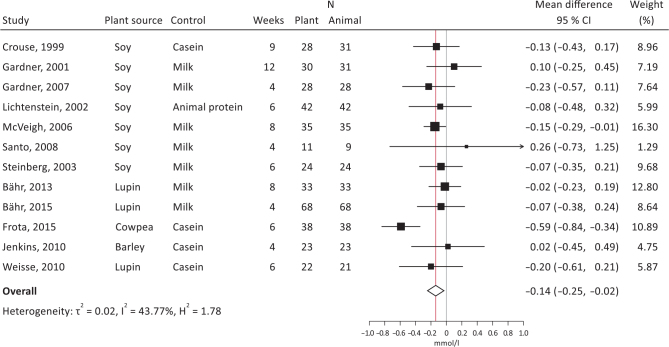
Meta-analysis of RCT studies of LDL-cholesterol. Forest plot showing mean differences with 95% CI in total cholesterol (mmol/l) by replacing animal protein with plant protein. The summary effect estimate (white diamond) was estimated by a restricted maximum likelihood random-effects model. RCT, randomized controlled trials; CT, confidence interval.

**Fig. 8:**
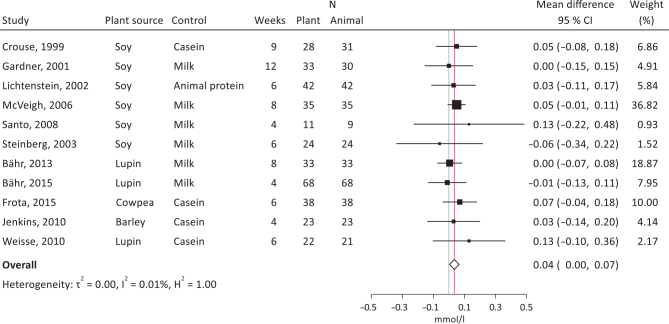
Meta-analysis of RCT studies of HDL-cholesterol. Forest plot showing mean differences with 95% CI in total cholesterol (mmol/l) by replacing animal protein with plant protein. The summary effect estimate (white diamond) was estimated by a restricted maximum likelihood random-effects model. RCT, randomized controlled trials; CT, confidence interval.

**Fig. 9:**
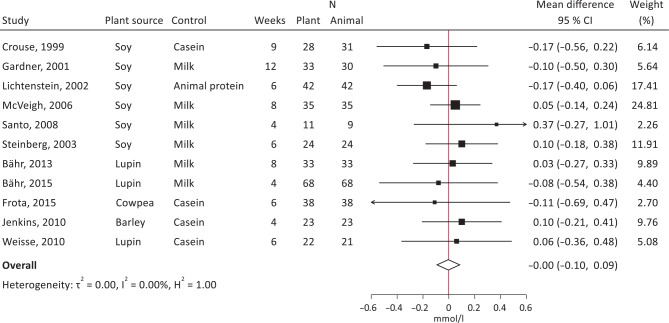
Meta-analysis of RCT studies of triacylglycerol. Forest plot showing mean differences with 95% CI in total cholesterol (mmol/l) by replacing animal protein with plant protein. The summary effect estimate (white diamond) was estimated by a restricted maximum likelihood random-effects model. RCT, randomized controlled trials; CT, confidence interval.

Both crossover and parallel studies were pooled in the meta-analyses. The summary effect sizes showed significantly decreased total cholesterol (Fig. 6; -0.11 mmol/L, 95% CI, -0.22, -0.01, *I*^2^ = 8.3%) and LDL-cholesterol (Fig. 7; -0.14 mmol/L, 95% CI, -0.25, -0.02, *I*^2^ = 43.8%), with plant protein interventions compared to animal protein, a borderline significantly increased HDL-cholesterol (Fig. 8; 0.04 mmol/L, 95% CI, 0.00, 0.07, *I*^2^ = 0.01%), but unsignificant effects on TG (Fig. 9; -0.00 mmol/L, 95% CI, -010, 0.09, *I*^2^ = 0.00%). It should be noted that Dent et al. (28) could not be meta-analyzed as results were only presented as *P*-values, and Gardner et al. (30) could only be included in the LDL-cholesterol meta-analysis.

In subsequent assessment, the meta-analyses of the RCTs were stratified by the plant protein source with subgroup analyses of soy vs. non-soy interventions (Supplementary file 3). No clear differences in blood lipids between the soy and the non-soy interventions in comparison to animal protein were observed.

Based on inspection of funnel plots (not shown), and Egger’s test for all meta-analyses including all intervention studies, we did not find evidence of publication bias in the form of small study-effects bias.

### Plant protein, blood pressure, blood glucose, and insulin concentration

Two studies (25, 26) investigated the impact of lupin protein or barley (32) and observed no effect on blood pressure compared to the animal protein (Tables 2 and 4). Three papers studied the effect of plant protein in comparison with animal protein on blood glucose (30, 35, 37) and one on fasting insulin (30), with no differences between the treatment groups. No meta-analyses were conducted for these outcomes, as the number of studies were insufficient.

### Substitution of animal protein with plant protein and CVD

Only one prospective cohort study (44) was retrieved that focused on the incidence of CHD using substitution model design (Tables 3 and 5). Although non-significant, a higher plant protein intake tended to be associated with a lower risk of CHD when consumed at the expense of animal protein. All four prospective studies (38, 39, 41, 42) with an isocaloric substitution of animal protein with plant protein showed lower risk of CVD mortality (Tables 3 and 5). Of these, Song et al. (41) found that substituting animal protein from processed or unprocessed red meat, fish, or dairy with plant protein was associated with lower CVD mortality. Budhathoki et al. (38) found that replacing animal protein from red meat (not from processed meat, chicken, egg, dairy, or fish) with plant protein reduced CVD mortality. Huang et al. (39) found that replacing total animal protein with plant protein was associated with lower mortality from CVD, heart disease, and stroke in both men and women. When separating on sources of animal protein, results remained for red meat, dairy, and egg, but replacing white meat protein with plant protein was only significantly associated with lower stroke mortality in men.

**Table 5:** Summary of findings from cohort studies

Author Year Population	Outcome	Exposure	Substitution of animal protein with plant protein	Conclusions	Overall risk of bias
**CVD**					
Budhathoki et al. 2019 Japan	CVD, heart disease and cerebrovascular disease mortality	Animal protein, plant protein; Mean (SD) intakes, expressed as percentage of total energy: Animal protein: 7.7 (2.7) Plant protein: 6.7 (1.4)	**Substituting 3 E% plant protein for animal protein**: HR (95% CI) **Red meat:** 0.58 (0.38–0.86) **Processed meat:** 0.58 (0.29–1.14) **Chicken:** 0.84 (0.50–1.42) **Egg:** 0.79 (0.57–1.11) **Dairy:** 0.82 (0.56–1.18) **Fish:** 0.86 (0.69–1.08)	Replacement of red or processed meat protein with plant protein was associated with a decreased risk of total, cancer-related, and CVD-related mortality. The study suggests that encouraging diets with higher plant-based protein intake may contribute to long-term health and longevity.	Moderate
Huang et al. 2020 USA	CVD, heart disease and stroke mortality	Median plant protein intake: Men: 26.9 g/d (14.4 g/1,000 kcal/d) Women: 21.6 g/d (14.9 g/1,000 kcal/d)	**Substituting 3 E% plant protein for animal protein** HR (95% CI) **Total animal protein** CVD Men: 0.89 (0.85–0.94) Women: 0.88 (0.82–0.94) Heart disease: Men: 0.91 (0.86–0.96) Women: 0.91 0.90 (0.84–0.98) Stroke Men: 0.78 (0.68–0.90) Women: 0.81 (0.70–0.94) **Red meat protein** CVD Men: 0.88 (0.83–0.93) Women: 0.82 (0.76–0.89) Heart disease Men: 0.89 (0.84–0.94) Women: 0.84 (0.77–0.92) Stroke Men: 0.79 (0.68–0.91) Women: 0.79 0.75 (0.63–0.89) **White meat protein** CVD Men: 0.95 (0.90–1.01) Women: 0.94 (0.87–1.02) Heart disease Men: 0.97 (0.91–1.03) Women: 0.97 (0.89–1.05) Stroke Men: 0.83 (0.71–0.96) Women: 0.90 (0.76–1.06)	Small but significant associations between higher intake of plant protein and lower overall and CVD mortality, with prominent inverse associations observed for replacement of egg protein and red meat protein with plant protein.	Moderate
			**Dairy protein** CVD Men: 0.89 (0.84–0.94) Women: 0.88 (0.82–0.95) Heart disease Men: 0.91 (0.86–0.97) Women: 0.92 (0.84–0.99) Stroke Men: 0.77 (0.66–0.89) Women: 0.80 (0.69–0.94) **Egg protein** CVD Men: 0.74 (0.67–0.82) Women: 0.72 (0.63–0.83) Heart disease Men: 0.76 (0.69–0.85) Women: 0.72 (0.62–0.85) Stroke Men: 0.67 (0.52–0.88) Women: 0.75 (0.55–1.03)		
Song et al. 2016 USA	CVD mortality	Animal protein, plant protein; ‘Percentage of total energy: Animal protein: 14%, Plant protein: 4%’	**Replacement of 3% energy from various animal protein sources with plant protein** HR (95% CI) **Processed red meat:** 0.61 (0.48–0.78) **Unprocessed red meat:** 0.83 (0.76–0.91) **Poultry:** 0.91 (0.83–1.00) **Fish:** 0.88 (0.80–0.97) **Egg:** 0.88 (0.75–1.04) **Dairy:** 0.89 (0.80–0.98)	Substitution of plant protein for animal protein, especially from processed red meat, may confer a substantial health benefit.	Moderate
Sun et al. 2021 USA	CVD mortality	Animal protein, plant protein Median percentage of total energy: Animal protein: 7.5% Plant protein: 3.5%	**Replacement of 5% of energy from animal protein with plant protein** HR (95% CI) CVD: 0.81 (0.72–0.92) (estimated from figure)	Substitution of animal protein with plant protein was associated with lower CVD mortality.	Moderate
Voortman et al. 2021	CHD incidence	Total protein, animal protein, and plant protein Mean (SD) Total protein in g/d 85.4 (23.9) Total protein in E% 16.3 (2.9) Plant protein in g/d 32.3 (11.9) Plant protein in E% 6.1 (1.3) Animal protein in g/d 53.0 (18.3) Animal protein in E% 10.2 (3.1)	**Replacement of 5% energy intake from animal protein with plant protein (and other macronutrients)** HR (95% CI) 0.69 (0.38–1.23)	Macronutrient composition was not significantly associated with CHD incidence or cardiometabolic risk factors.	Moderate
**T2D**					
Virtanen et al. 2017 Finland	Incident T2D	Total protein, animal protein, and plant protein Mean (SD) g/day: Total: 92.9 (14.4) Animal: 64.8 (15.4) Vegetable: 25.8 (6.0)	Replacement of 1% energy from different animal protein sources with plant protein: HR (95% CI) **Animal protein:** 0.81 (0.67–0.98) **Total meat protein:** 0.83 (0.68–1.01) **Red meat:** 0.82 (0.67–1.01) **Processed red meat:** 0.80 (0.64–0.99) **Unprocessed red meat**: 0.83 (0.68–1.01) **Fish:** 0.85 (0.69–1.04) **Dairy**: 0.79 (0.65–0.97), **Non-fermented dairy:** 0.79 (0.64–0.97) **Fermented dairy:** 0.79 (0.65–0.97) **Egg**: 1.11 (0.68–1.82)	Favoring protein from plant sources and eggs over other animal sources may be beneficial in the prevention of T2D.	Moderate
Malik et al. 2016 USA	Incident T2D	Total protein, animal protein, and plant protein Mean percentages of energy intake: NHS: Total protein: 18.1% Animal protein: 15.1% Vegetable protein: 5% NHS II: Total: 18.9% Animal: 13.7% Vegetable protein: 7.3% HPFS: Total: 18.2% Animal: 13.0% Vegetable protein: 5.1%	**Substitution of vegetable protein for animal protein:** HR (95% CI) 0.77 (0.70–0.84)	Substituting vegetable protein for animal protein was associated with reduced risk of T2D.	Moderate

CHD, coronary heart disease; CVD, cardiovascular disease; T2D, type 2 diabetes; HR, hazard ratio; CI, confidence interval.

### Substitution of animal protein with plant protein and T2D incidence

Only two papers (40, 43) fulfilled our inclusion criteria for T2D incidence, and both showed associations with reduced T2D incidence with isocaloric substitution of animal protein with plant protein (Tables 3 and 5). Virtanen et al. (43) also showed that replacing any animal protein except for protein from eggs with energy from plant protein was associated with a 14–20% decreased risk of T2D, although not all associations reached statistical significance.

### Certainty in the evidence

The evidence for a favorable association between plant protein intake in comparison to animal protein and CVD mortality was considered *limited-suggestive* based on consistent results from cohort studies with moderate risk of bias, supported by evidence of biological plausibility from the RCTs. The corresponding evidence for T2D incidence was considered *limited, suggestive*, while the few available RCT studies on blood glucose and insulin did not support an effect.

## Discussion

### Summary of key findings

This SR summarizes both RCTs and cohort studies for whether substituting plant protein for animal protein is associated with lower risk of CVD and T2D or lower levels of cardiometabolic risk factors. While the cohort studies reported associations with decreased risks of CVD and T2D in substitution models of animal protein with plant protein, the biological plausibility based on the RCTs was supported for CVD alone. Evidence was considered *limited-suggestive* for reduced CVD mortality and T2D, when replacing animal protein with plant protein.

### Strengths and limitation of review

A strength of this review is that we followed established processes for undertaking robust SRs. The NNR 2022 Committee established criteria for the prioritization and selection of a SR topic (10). We developed and registered a detailed protocol before undertaking the review, which improved transparency of the review process. We searched four foremost electronic databases, which cover most of the literature in medicine and public health, why it is unlikely that we may have missed any relevant literature. Moreover, the review processes were thoroughly implemented, with independent assessments taken at each stage of the process, including literature screening and data extraction.

One-third of the RCTs was graded as having a high risk of bias, especially due to deviations from the intended intervention, another third was graded having some concerns regarding risk of bias, mainly arising from the randomization process. Additional limitations include the habitual diets in the RCTs, which may have affected the ability to detect effects of the intervention. Moreover, the animal protein in the RCTs was milk protein or casein, which may not be totally representative for animal protein sources. Among the RCTs, eight investigated soy protein (27–31, 33–36) and five other plant proteins, including other legumes (25, 26, 29, 37) and grains (32). Although overall results were not different for the different sources of plant protein, it could be worth in future studies to focus on other legumes and grains instead of soy. We did not find RCTs comparing other sources of plant protein intake, than those above mentioned, to animal protein intake in our search.

All included cohort studies were graded as having a moderate risk of bias, which may constitute a limitation of the underlying evidence. We extracted studies that reported on plant protein intake in relation to animal protein intake, but this may, however, not cover all possible sources of plant protein. Most of the studies were prone to limitations inherent in many observational epidemiologic studies – the starting time of the exposure, method of assessment of dietary intake as it was based on self-reported data (which, in addition, is usually done once at baseline), and inadequate adjustment for confounding factors during the long follow-up, thereby given a possibility for residual/unmeasured confounding across the reported estimates in the studies.

## Comparison with other SRs

We retrieved three previous SRs and meta-analyses related to the comparison of animal protein intake to plant protein intake or other diets with blood lipids as outcomes in RCTs settings (45–47). Guasch-Ferré et al. (45) included 36 RCTs, comparing diets with red meat to diets that replaced red meat with a variety of foods. They concluded that substituting red meat with high-quality plant protein sources, but not with fish or low-quality carbohydrates, leads to more favorable changes in blood lipids and lipoproteins. Li et al. (46) included 104 RCTs, also including individuals with, e.g., T2D and renal disease, comparing the effect of plant protein in substitution for animal protein on blood lipids. They concluded that substitution of plant protein for animal protein decreases LDL-cholesterol and non-HDL cholesterol. Zhao et al. (47) focused on effects of plant protein and animal protein on lipid profile as well as body weight and body mass index in patients with confirmed hypercholesterolemia. They concluded that compared with animal protein, the consumption of plant protein could improve lipid profile in patients with hypercholesterolemia. Our results support the results from previous SRs, even though we only included soy intake with low concentrations of isoflavones and subjects with normal serum cholesterol concentrations or mild hypercholesterolemia, which was reflected in the low number of studies included.

We found two previous SRs focused on protein intake, including plant protein intake and risk of CVD mortality (6, 7). Naghshi et al. (6) concluded that higher intake of plant protein was associated with a lower risk of CVD mortality, whereas there was no association of total protein or animal protein with the risk of CVD mortality. Qi et al. reported (7) that higher plant protein intake (but not total protein) was associated with a reduced risk of CVD related- and all-cause mortality. In conclusion, our results seem to be in line with these two SRs.

A previous SR and meta-analysis showed that total protein and animal protein intake was associated with a higher risk of T2D in both males and females, and that plant protein decreased the risk of T2D in females. These associations were also dependent on the food source, as e.g. red meat and processed meat were risk factors of T2D, while soy, dairy, and dairy products were protective against T2D (48). Our results point in the same direction, but we included fewer cohort studies, as the exposure was defined as substitution of animal protein with plant protein.

Altogether we found six recent SRs that could be considered comparable to the current paper (5, 6, 45–48). The inclusion criteria were overall not exactly the same as ours as we did not include interventions with soy containing high or medium levels of isoflavones, in contrast to the previous reviews. In addition, we included only prospective cohorts, which compared substitution of animal protein with plant protein, i.e. substitution analyses. These differences in inclusion criteria lead to a lower number of included studies in comparison to previous SRs.

### Interpretation and implications of findings

The intervention studies showed significantly, albeit only small lowering of total cholesterol and LDL-cholesterol along with higher HDL-cholesterol as a result of plant protein intake in comparison with animal protein intake. Soy, which has been studied extensively, may have a favorable effect on blood lipids, since it contains or can be fortified with high amount of isoflavones, which are known to have these effects (16). Although the magnitudes of the differences in cholesterol levels were small, they may be relevant in a life-course population perspective. The results of the cohort studies indicated an association between substitution of animal protein with plant protein on the risk of CVD and T2D. In comparison with most animal protein sources, plant protein sources contain less saturated fat and no cholesterol and more monounsaturated and polyunsaturated fat, fiber, antioxidants, polyphenols, and other bioactive compounds (49). Other mechanisms have also been suggested, i.e., related to amino acid metabolism. Lysine, which is more prevalent in animal proteins, has been shown to increase cholesterol levels in animal models, whereas arginine, which is found more in plant proteins, has been found to have the opposite effect (47).

## Conclusion

We found *limited-suggestive* evidence that substitution of animal protein with plant protein may decrease the risk of CVD mortality and T2D incidence. Protective effects seen in RCTs on established risk factors for CVD supported the evidence from observational studies. Replacement of animal protein with plant protein for sustainability may also be considered as a public health strategy to lower the risk of CVD and T2D.
