# Supplementary material for: Animal versus plant-based protein and risk of cardiovascular disease and type 2 diabetes: a systematic review of randomized controlled trials and prospective cohort studies
Source: Food Nutr Res. 2023 Mar 28;67:10.29219/fnr.v67.9003. doi: 10.29219/fnr.v67.9003 (PMC10084508; doi:10.29219/fnr.v67.9003)
Supplement: Supplementary file 1 [file FNR-67-9003-s001.docx]

Supplement 1. Documentation of literature search

The following data bases were searched

| Database | Number of retrieved references |
| --- | --- |
| MEDLINE (Ovid) | 2261 |
| Embase (Ovid) | 3627 |
| Cochrane Central Register of controlled Trials | 2015 |
| Scopus | 7187 |
| Number of references before deduplication: | 15090 |
| Number of references after deduplication: | 9950 |
|  |  |

| Database(s): **Ovid MEDLINE(R) and Epub Ahead of Print, In-Process, In-Data-Review & Other Non-Indexed Citations and Daily**1946 to March 25, 2021 Search Strategy:   \| **#** \| **Searches** \| **Results** \| \| --- \| --- \| --- \| \| 1 \| exp Plant Proteins, Dietary/ \| 6351 \| \| 2 \| ((bean? or chickpea? or fruit? or grain? or legume? or lentil? or nut? or plant? or pea or peas or pulse? or seed? or soy* or vegetable?) adj4 protein?).ti,ab,kf. \| 36727 \| \| 3 \| ((consum* or diet* or eat or eating or fed or feed or food? or ingest* or intake* or nutrition or nutrient? or source?) adj3 protein?).ti,ab,kf. \| 54203 \| \| 4 \| (bean? or chickpea? or fruit? or grain? or legume? or lentil? or nut? or plant? or pea or peas or pulse? or seed? or soy* or vegetable?).ti,ab,kf. \| 1098414 \| \| 5 \| 3 and 4 \| 11074 \| \| 6 \| 1 or 2 or 5 \| 44777 \| \| 7 \| Cardiovascular Diseases/ \| 154377 \| \| 8 \| Atherosclerosis/ \| 37739 \| \| 9 \| exp Myocardial Infarction/ \| 178917 \| \| 10 \| exp Stroke/ \| 141848 \| \| 11 \| exp Coronary Disease/ \| 221461 \| \| 12 \| exp Coronary Artery Bypass/ \| 53735 \| \| 13 \| ((ACA or anterior cerebral artery or anterior cerebral circulation or anterior choroidal artery or brain or brain stem or brainstem or brain venous or cerebral or heart or heubner* artery or MCA or middle cerebral artery or myocardial or PCA or posterior cerebral artery or posterior choroidal artery or subcortical) adj2 infarct*).ti,ab,kf. \| 234447 \| \| 14 \| ((anterior cerebral artery or basilar or benedict or claude or coronary-subclavian steal or dorsolateral medullary or foville or lateral bulbar or lateral medullary or middle cerebral artery or millard-gublar or posterior cerebral artery or posterior inferior cerebellar artery or wallenberg* or weber) adj2 syndrome*).ti,ab,kf. \| 3489 \| \| 15 \| ((brain vascular or cerebrovascular) adj2 accident*).ti,ab,kf. \| 7511 \| \| 16 \| ((coronary artery or aortocoronary) adj2 bypass*).ti,ab,kf. \| 44952 \| \| 17 \| (coronary adj3 (aneurysm* or arterioscleros#s or artery anastomos#s or disease* or occlusion* or restenos#s or stenos#s or syndrome* or thrombos#s or vasospasm*)).ti,ab,kf. \| 217252 \| \| 18 \| (apoplex* or atherogenesis or atheroscleros#s or cardiogenic shock or heart attack* or middle cerebral artery thrombosis or stroke*).ti,ab,kf. \| 402495 \| \| 19 \| ((cardio* or cerebral* or cerebro* or coronary or CVD or heart* or myocardial) adj3 (death? or mortalit*)).ti,ab,kf. \| 91023 \| \| 20 \| or/7-19 \| 1058785 \| \| 21 \| 6 and 20 \| 778 \| \| 22 \| exp Diabetes Mellitus, Type 2/ \| 139074 \| \| 23 \| exp Insulin Resistance/ \| 86036 \| \| 24 \| C-Peptide/ \| 8519 \| \| 25 \| Glucose Intolerance/ \| 8960 \| \| 26 \| Glycated Hemoglobin A/ \| 36300 \| \| 27 \| Blood Glucose/ \| 167843 \| \| 28 \| Hyperglycemia/ \| 28729 \| \| 29 \| Blood Pressure/ \| 278284 \| \| 30 \| Hypertension/ \| 238901 \| \| 31 \| Lipids/ \| 115548 \| \| 32 \| exp Triglycerides/ \| 78164 \| \| 33 \| exp Apolipoproteins/ \| 47129 \| \| 34 \| Cholesterol/ \| 121065 \| \| 35 \| Cholesterol, HDL/ \| 28856 \| \| 36 \| Cholesterol, LDL/ \| 28275 \| \| 37 \| Cholesterol, VLDL/ \| 2025 \| \| 38 \| (diabet* adj3 ("2" or "type II" or Adult-Onset or Non Insulin or NonInsulin)).ti,ab,kf. \| 173974 \| \| 39 \| (DM2 or NIDDM or IIDM or MODY or T2DM).ti,ab,kf. \| 33678 \| \| 40 \| (blood adj2 (glucose or sugar*)).ti,ab,kf. \| 91010 \| \| 41 \| (lipid* adj2 (blood or level or profile*)).ti,ab,kf. \| 54416 \| \| 42 \| (blood pressure or cardiometabolic syndrome* or C-peptide or cholesterol or HDL or LDL or VLDL or connecting peptide or diastolic pressure or dysmetabolic syndrome* or glucose intolerance* or HOMA-IR or hyperglycemia* or hypertension or insulin resistance or insulin sensitivity or metabolic syndrome* or metabolic cardiovascular syndrome* or pulse pressure or reaven syndrome X or systolic pressure).ti,ab,kf. \| 1016611 \| \| 43 \| ((glycated or glycosylated) adj2 (haemoglobin* or hemoglobin*)).ti,ab,kf. \| 23504 \| \| 44 \| (glycohemoglobin A or Hb A1 or HbA1 or Hb A1a-1 or Hb A1a-2 or Hb A1a+b or Hb A1b or Hb A1c or HbA1c or "hemoglobin A(1)" or hemoglobin A1C).ti,ab,kf. \| 45267 \| \| 45 \| (Apo-B or ApoA or ApoA-II or Apo A-V or Apo A1 or Apo A2 or Apo A5 or APOA5 or Apo AI or ApoB or ApoB48 or ApoC or Apo C or ApoC2 or Apo D or ApoD or ApoE or Apo E or APOE-epsilon* or ApoE2 or Apo E2 or Apo E3 or ApoE3 or Apo E4 or ApoE4 or ApoL or ApoL1 or apolipoprotein* or apoprotein* or enzactin or glycerol trioleate or proapolipoprotein* or triacetin or triacetyl-glycerol* or triacetylglycerol* or triacylglycerol* or trielaidin or triglyceride* or trioleate-glycerin or triolein or trioleoylglycerol or trioleyl glycerol*).ti,ab,kf. \| 189932 \| \| 46 \| or/22-45 \| 1600829 \| \| 47 \| 6 and 46 \| 3594 \| \| 48 \| 21 or 47 \| 3812 \| \| 49 \| 48 not (animals not humans).sh. \| 2692 \| \| 50 \| 49 not (Editorial/ or Letter/ or Review/ or Systematic Review/ or Meta-Analysis/) \| 2098 \| |
| --- | --- | --- | --- | --- | --- | --- | --- | --- | --- | --- | --- | --- | --- | --- | --- | --- | --- | --- | --- | --- | --- | --- | --- | --- | --- | --- | --- | --- | --- | --- | --- | --- | --- | --- | --- | --- | --- | --- | --- | --- | --- | --- | --- | --- | --- | --- | --- | --- | --- | --- | --- | --- | --- | --- | --- | --- | --- | --- | --- | --- | --- | --- | --- | --- | --- | --- | --- | --- | --- | --- | --- | --- | --- | --- | --- | --- | --- | --- | --- | --- | --- | --- | --- | --- | --- | --- | --- | --- | --- | --- | --- | --- | --- | --- | --- | --- | --- | --- | --- | --- | --- | --- | --- | --- | --- | --- | --- | --- | --- | --- | --- | --- | --- | --- | --- | --- | --- | --- | --- | --- | --- | --- | --- | --- | --- | --- | --- | --- | --- | --- | --- | --- | --- | --- | --- | --- | --- | --- | --- | --- | --- | --- | --- | --- | --- | --- | --- | --- | --- | --- | --- | --- | --- |

2. Embase

| Interface: embase.com  Date of Search: 25 March 2021  Number of hits: 3,350  Comment: Emtree is the controlled vocabulary in Embase | Field labels   - /exp = exploded Emtree term - /de = non exploded Emtree term - ti,ab,kw = title, abstract and author keywords - NEAR/x = within x words, regardless of order - * = truncation of word for alternate endings |
| --- | --- |
| (('plant protein'/exp OR (((bean$ OR chickpea$ OR fruit$ OR grain$ OR legume$ OR lentil$ OR nut$ OR plant$ OR pea OR peas OR pulse$ OR seed$ OR soy* OR vegetable$) NEAR/4 protein$):ti,ab,kw) OR ((((consum* OR diet* OR eat OR eating OR fed OR feed OR food$ OR ingest* OR intake* OR nutrition OR nutrient$ OR source$) NEAR/3 protein$):ti,ab,kw) AND (bean$:ti,ab,kw OR chickpea$:ti,ab,kw OR fruit$:ti,ab,kw OR grain$:ti,ab,kw OR legume$:ti,ab,kw OR lentil$:ti,ab,kw OR nut$:ti,ab,kw OR plant$:ti,ab,kw OR pea:ti,ab,kw OR peas:ti,ab,kw OR pulse$:ti,ab,kw OR seed$:ti,ab,kw OR soy*:ti,ab,kw OR vegetable$:ti,ab,kw))) AND ('cardiovascular disease'/de OR 'atherosclerosis'/de OR 'heart infarction'/exp OR 'cerebrovascular accident'/exp OR 'coronary artery disease'/exp OR 'coronary artery bypass graft'/de OR (((aca OR 'anterior cerebral artery' OR 'anterior cerebral circulation' OR 'anterior choroidal artery' OR brain OR 'brain stem' OR brainstem OR 'brain venous' OR cerebral OR heart OR 'heubner* artery' OR mca OR 'middle cerebral artery' OR myocardial OR pca OR 'posterior cerebral artery' OR 'posterior choroidal artery' OR subcortical) NEAR/2 infarct*):ti,ab,kw) OR ((('anterior cerebral artery' OR basilar OR benedict OR claude OR 'coronary-subclavian steal' OR 'dorsolateral medullary' OR foville OR 'lateral bulbar' OR 'lateral medullary' OR 'middle cerebral artery' OR 'millard-gublar' OR 'posterior cerebral artery' OR 'posterior inferior cerebellar artery' OR wallenberg* OR weber) NEAR/2 syndrome*):ti,ab,kw) OR ((('brain vascular' OR cerebrovascular) NEAR/2 accident*):ti,ab,kw) OR ((('coronary artery' OR aortocoronary) NEAR/2 bypass*):ti,ab,kw) OR ((coronary NEAR/3 (aneurysm* OR arterioscleros?s OR 'artery anastomos?s' OR disease* OR occlusion* OR restenos?s OR stenos?s OR syndrome* OR thrombos?s OR vasospasm*)):ti,ab,kw) OR apoplex*:ti,ab,kw OR atherogenesis:ti,ab,kw OR atheroscleros?s:ti,ab,kw OR 'cardiogenic shock':ti,ab,kw OR 'heart attack*':ti,ab,kw OR 'middle cerebral artery thrombosis':ti,ab,kw OR stroke*:ti,ab,kw OR (((cardio* OR cerebral* OR cerebro* OR coronary OR cvd OR heart* OR myocardial) NEAR/3 (death$ OR mortalit*)):ti,ab,kw)) OR (('plant protein'/exp OR (((bean$ OR chickpea$ OR fruit$ OR grain$ OR legume$ OR lentil$ OR nut$ OR plant$ OR pea OR peas OR pulse$ OR seed$ OR soy* OR vegetable$) NEAR/4 protein$):ti,ab,kw) OR ((((consum* OR diet* OR eat OR eating OR fed OR feed OR food$ OR ingest* OR intake* OR nutrition OR nutrient$ OR source$) NEAR/3 protein$):ti,ab,kw) AND (bean$:ti,ab,kw OR chickpea$:ti,ab,kw OR fruit$:ti,ab,kw OR grain$:ti,ab,kw OR legume$:ti,ab,kw OR lentil$:ti,ab,kw OR nut$:ti,ab,kw OR plant$:ti,ab,kw OR pea:ti,ab,kw OR peas:ti,ab,kw OR pulse$:ti,ab,kw OR seed$:ti,ab,kw OR soy*:ti,ab,kw OR vegetable$:ti,ab,kw))) AND ('non insulin dependent diabetes mellitus'/de OR 'insulin resistance'/de OR 'c peptide'/de OR 'glucose intolerance'/de OR 'hemoglobin a1c'/de OR 'glucose blood level'/de OR 'hyperglycemia'/de OR 'blood pressure'/de OR 'hypertension'/de OR 'lipid blood level'/exp OR 'apolipoprotein'/exp OR 'high density lipoprotein cholesterol'/de OR 'low density lipoprotein cholesterol'/de OR 'very low density lipoprotein cholesterol'/de OR ((diabet* NEAR/3 ('2' OR 'type ii' OR 'adult-onset' OR 'non insulin' OR noninsulin)):ti,ab,kw) OR dm2:ti,ab,kw OR niddm:ti,ab,kw OR iidm:ti,ab,kw OR mody:ti,ab,kw OR t2dm:ti,ab,kw OR ((blood NEAR/2 (glucose OR sugar*)):ti,ab,kw) OR ((lipid* NEAR/2 (blood OR level OR profile*)):ti,ab,kw) OR 'blood pressure':ti,ab,kw OR 'cardiometabolic syndrome*':ti,ab,kw OR 'c-peptide':ti,ab,kw OR cholesterol:ti,ab,kw OR hdl:ti,ab,kw OR ldl:ti,ab,kw OR vldl:ti,ab,kw OR 'connecting peptide':ti,ab,kw OR 'diastolic pressure':ti,ab,kw OR 'dysmetabolic syndrome*':ti,ab,kw OR 'glucose intolerance*':ti,ab,kw OR 'homa-ir':ti,ab,kw OR hyperglycemia*:ti,ab,kw OR hypertension:ti,ab,kw OR 'insulin resistance':ti,ab,kw OR 'insulin sensitivity':ti,ab,kw OR 'metabolic syndrome*':ti,ab,kw OR 'metabolic cardiovascular syndrome*':ti,ab,kw OR 'pulse pressure':ti,ab,kw OR 'reaven syndrome x':ti,ab,kw OR 'systolic pressure':ti,ab,kw OR (((glycated OR glycosylated) NEAR/2 (haemoglobin* OR hemoglobin*)):ti,ab,kw) OR 'glycohemoglobin a':ti,ab,kw OR 'hb a1':ti,ab,kw OR 'hba1':ti,ab,kw OR 'hb a1a-1':ti,ab,kw OR 'hb a1a-2':ti,ab,kw OR 'hb a1a+b':ti,ab,kw OR 'hb a1b':ti,ab,kw OR 'hb a1c':ti,ab,kw OR 'hba1c':ti,ab,kw OR 'hemoglobin a(1)':ti,ab,kw OR 'hemoglobin a1c':ti,ab,kw OR 'apo-b':ti,ab,kw OR 'apoa':ti,ab,kw OR 'apoa-ii':ti,ab,kw OR 'apo a-v':ti,ab,kw OR 'apo a1':ti,ab,kw OR 'apo a2':ti,ab,kw OR 'apo a5':ti,ab,kw OR 'apoa5':ti,ab,kw OR 'apo ai':ti,ab,kw OR 'apob':ti,ab,kw OR 'apob48':ti,ab,kw OR 'apoc':ti,ab,kw OR 'apo c':ti,ab,kw OR 'apoc2':ti,ab,kw OR 'apo d':ti,ab,kw OR 'apod':ti,ab,kw OR 'apoe':ti,ab,kw OR 'apo e':ti,ab,kw OR 'apoe-epsilon*':ti,ab,kw OR 'apoe2':ti,ab,kw OR 'apo e2':ti,ab,kw OR 'apo e3':ti,ab,kw OR 'apoe3':ti,ab,kw OR 'apo e4':ti,ab,kw OR 'apoe4':ti,ab,kw OR 'apol':ti,ab,kw OR 'apol1':ti,ab,kw OR apolipoprotein*:ti,ab,kw OR apoprotein*:ti,ab,kw OR enzactin:ti,ab,kw OR 'glycerol trioleate':ti,ab,kw OR proapolipoprotein*:ti,ab,kw OR triacetin:ti,ab,kw OR 'triacetyl-glycerol*':ti,ab,kw OR triacetylglycerol*:ti,ab,kw OR triacylglycerol*:ti,ab,kw OR trielaidin:ti,ab,kw OR triglyceride*:ti,ab,kw OR 'trioleate-glycerin':ti,ab,kw OR triolein:ti,ab,kw OR trioleoylglycerol:ti,ab,kw OR 'trioleyl glycerol*':ti,ab,kw)))  NOT    ([animals]/lim NOT [humans]/lim) AND ([article]/lim OR [article in press]/lim OR [erratum]/lim) | |

| Interface: Wiley  Date of Search: 28 March 2021  Number of hits: 1876 | Field labels   - ti,ab,kw = title, abstract and author keywords - pt = publication type - NEAR/x = within x words, regardless of order - * = truncation of word for alternate endings |
| --- | --- |
| \| **#** \| **Searches** \| **Results** \| \| --- \| --- \| --- \| \| 1 \| [mh ”Plant Proteins, Dietary”] \| 489 \| \| 2 \| ((bean* OR chickpea* OR fruit* OR grain* OR legume* OR lentil* OR nut* OR plant* OR pea OR peas OR pulse* OR seed* OR soy* OR vegetable*) NEAR/4 protein*):ab,ti,kw \| 3723 \| \| 3 \| ((consum* OR diet* OR eat OR eating OR fed OR feed OR food* OR ingest* OR intake* OR nutrition OR nutrient* OR source*) NEAR/3 protein*):ab,ti,kw \| 8877 \| \| 4 \| (bean* OR chickpea* OR fruit* OR grain* OR legume* OR lentil* OR nut* OR plant* OR pea OR peas OR pulse* OR seed* OR soy* OR vegetable*):ab,ti,kw \| 104975 \| \| 5 \| #3 AND #4 \| 4801 \| \| 6 \| #1 OR #2 OR #5 \| 6581 \| \| 7 \| [mh ^"Cardiovascular Diseases"] \| 8335 \| \| 8 \| [mh ^Atherosclerosis] \| 1384 \| \| 9 \| [mh "Myocardial Infarction"] \| 11251 \| \| 10 \| [mh Stroke] \| 10142 \| \| 11 \| [mh "Coronary Disease"] \| 13964 \| \| 12 \| [mh "Coronary Artery Bypass"] \| 5471 \| \| 13 \| (((ACA OR "anterior cerebral artery" OR "anterior cerebral circulation" OR "anterior choroidal artery" OR brain OR "brain stem" OR brainstem OR "brain venous" OR cerebral OR heart OR (heubner* NEXT artery) OR MCA OR "middle cerebral artery" OR myocardial OR PCA OR "posterior cerebral artery" OR "posterior choroidal artery" OR subcortical) NEAR/2 infarct*)):ab,ti,kw \| 39458 \| \| 14 \| (("anterior cerebral artery" OR basilar OR benedict OR claude OR "coronary-subclavian steal" OR "dorsolateral medullary" OR foville OR "lateral bulbar" OR "lateral medullary" OR "middle cerebral artery" OR "millard-gublar" OR "posterior cerebral artery" OR "posterior inferior cerebellar artery" OR wallenberg* OR weber) NEAR/2 syndrome*):ab,ti,kw \| 44 \| \| 15 \| (("brain vascular" OR cerebrovascular) NEAR/2 accident*):ab,ti,kw \| 13385 \| \| 16 \| (("coronary artery" OR aortocoronary) NEAR/2 bypass*):ab,ti,kw \| 12146 \| \| 17 \| (coronary NEAR/3 (aneurysm* OR arterioscleros?s OR (artery NEXT anastomos?s) OR disease* OR occlusion* OR restenos?s OR stenos?s OR syndrome* OR thrombos?s OR vasospasm*)):ab,ti,kw \| 36520 \| \| 18 \| (apoplex* OR atherogenesis OR atheroscleros?s OR "cardiogenic shock" (heart NEXT attack*) OR "middle cerebral artery thrombosis" OR stroke*):ab,ti,kw \| 67861 \| \| 19 \| ((cardio* OR cerebral* OR cerebro* OR coronary OR CVD OR heart* OR myocardial) NEAR/3 (death* OR mortalit*)):ab,ti,kw \| 22842 \| \| 20 \| #7 OR #8 OR #9 OR #10 OR #11 OR #12 OR #13 OR #14 OR #15 OR #16 OR #17 OR #18 OR #19 \| 134050 \| \| 21 \| #6 AND #20 \| 371 \| \| 22 \| [mh "Diabetes Mellitus, Type 2"] \| 18012 \| \| 23 \| [mh "Insulin Resistance"] \| 6412 \| \| 24 \| [mh ^"C-Peptide"] \| 1251 \| \| 25 \| [mh ^"Glucose Intolerance"] \| 1145 \| \| 26 \| [mh ^"Glycated Hemoglobin A"] \| 5790 \| \| 27 \| [mh ^"Blood Glucose"] \| 16193 \| \| 28 \| [mh ^Hyperglycemia] \| 1941 \| \| 29 \| [mh ^"Blood Pressure"] \| 26752 \| \| 30 \| [mh ^Hypertension] \| 17762 \| \| 31 \| [mh ^Lipids] \| 6409 \| \| 32 \| [mh Triglycerides] \| 6371 \| \| 33 \| [mh Apolipoproteins] \| 2017 \| \| 34 \| [mh ^Cholesterol] \| 5971 \| \| 35 \| [mh ^"Cholesterol, HDL"] \| 3732 \| \| 36 \| [mh ^"Cholesterol, LDL"] \| 4781 \| \| 37 \| [mh ^"Cholesterol, VLDL"] \| 249 \| \| 38 \| (diabet* NEAR/3 ("2" OR "type II" OR "Adult-Onset" OR "Non Insulin" OR NonInsulin)):ab,ti,kw \| 45162 \| \| 39 \| (DM2 OR NIDDM OR IIDM OR MODY OR T2DM):ab,ti,kw \| 7934 \| \| 40 \| (blood NEAR/2 (glucose OR sugar*)):ab,ti,kw \| 41262 \| \| 41 \| (lipid* NEAR/2 (blood OR level OR profile*)):ab,ti,kw \| 19610 \| \| 42 \| ("blood pressure" OR (cardiometabolic NEXT syndrome*) OR "C-peptide" OR cholesterol OR HDL OR LDL OR VLDL OR "connecting peptide" OR "diastolic pressure" OR (dysmetabolic NEXT syndrome*) OR (glucose NEXT intolerance*) OR "HOMA-IR" OR hyperglycemia* OR hypertension OR "insulin resistance" OR "insulin sensitivity" OR (metabolic NEXT syndrome*) OR ("metabolic cardiovascular" NEXT syndrome*) OR "pulse pressure" OR "reaven syndrome X" OR "systolic pressure"):ab,ti,kw \| 175780 \| \| 43 \| ((glycated OR glycosylated) NEAR/2 (haemoglobin* OR hemoglobin*)):ab,ti,kw \| 10972 \| \| 44 \| ("glycohemoglobin A" OR "Hb A1" OR "HbA1" OR "Hb A1a-1" OR "Hb A1a-2" OR "Hb A1a+b" OR "Hb A1b" OR "Hb A1c" OR "HbA1c" OR "hemoglobin A(1)" OR "hemoglobin A1C"):ab,ti,kw \| 22145 \| \| 45 \| ("Apo-B" OR ApoA OR "ApoA-II" OR "Apo A-V" OR "Apo A1" OR "Apo A2" OR "Apo A5" OR APOA5 OR "Apo AI" OR ApoB OR ApoB48 OR ApoC OR "Apo C" OR ApoC2 OR "Apo D" OR ApoD OR ApoE OR "Apo E" OR "APOE-epsilon*" OR ApoE2 OR "Apo E2" OR "Apo E3" OR ApoE3 OR "Apo E4" OR ApoE4 OR ApoL OR ApoL1 OR apolipoprotein* OR apoprotein* OR enzactin OR "glycerol trioleate" OR proapolipoprotein* OR triacetin OR "triacetyl-glycerol*" OR triacetylglycerol* OR triacylglycerol* OR trielaidin OR triglyceride* OR "trioleate-glycerin" OR triolein OR trioleoylglycerol OR (trioleyl NEXT glycerol*):ab,ti,kw \| 29166 \| \| 46 \| #22 OR #23 OR #24 OR #25 OR #26 OR #27 OR #28 OR #29 OR #30 OR #31 OR #32 OR #33 OR #34 OR #35 OR #36 OR #37 OR #38 OR #39 OR #40 OR #41 OR #42 OR #43 OR #44 OR #45 \| 221572 \| \| 47 \| #6 AND #46 \| 2106 \| \| 48 \| #21 OR #47 \| 2195 \| \| 49 \| ("Conference abstract" OR "Conference paper" OR "Conference review" OR Editorial OR Letter OR Meta-analysis OR Note OR Review OR "Short Survey" OR "Systematic review" OR Book OR "Book Chapter"):pt \| 221185 \| \| 50 \| #48 NOT #49 \| 1894 \| \| 51 \| #50 in Trials \| 1876 \| \|  \|  \|  \| \|  \|  \|  \| | |

4. Scopus

| Interface: Elsevier  Date of Search: 25 March 2021  Number of hits: 6,588  Comment: | Field labels   - TITLE-ABS-KEY = title, abstract and author keywords - W/x = within x words, regardless of order - = truncation of word for alternate endings |
| --- | --- |
| ((TITLE-ABS-KEY(((ACA or "anterior cerebral artery" or "anterior cerebral circulation" or "anterior choroidal artery" or brain or "brain stem" or brainstem or "brain venous" or cerebral or heart or "heubner* artery" or MCA or "middle cerebral artery" or myocardial or PCA or "posterior cerebral artery" or "posterior choroidal artery" or subcortical) W/1 infarct*)))  OR (TITLE-ABS-KEY((("anterior cerebral artery" or basilar or benedict or claude or "coronary-subclavian steal" or "coronary subclavian steal" or "dorsolateral medullary" or foville or "lateral bulbar" or "lateral medullary" or "middle cerebral artery" or "millard-gublar" or "millard gublar" or "posterior cerebral artery" or "posterior inferior cerebellar artery" or wallenberg* or weber) W/1 syndrome*)))  OR (TITLE-ABS-KEY((("brain vascular" or cerebrovascular) W/1 accident*)))  OR (TITLE-ABS-KEY((("coronary artery" or aortocoronary) W/1 bypass*)))  OR (TITLE-ABS-KEY((coronary W/2 (aneurysm* or arteriosclerosis or "artery anastomosis" or disease* or occlusion* or restenosis or stenosis or syndrome* or thrombosis or vasospasm*))))  OR (TITLE-ABS-KEY((apoplex* or atherogenesis or atherosclerosis or "cardiogenic shock" or "heart attack*" or "middle cerebral artery thrombosis" or stroke*)))  OR (TITLE-ABS-KEY(((cardio* or cerebral* or cerebro* or coronary or CVD or heart* or myocardial) W/2 (death* or mortalit*))))  OR (TITLE-ABS-KEY((diabet* W/2 ("2" or "type II" or "Adult-Onset" or "Adult Onset" or "Non Insulin" or NonInsulin))))  OR (TITLE-ABS-KEY((DM2 or NIDDM or IIDM or MODY or T2DM)))  OR (TITLE-ABS-KEY((blood W/1 (glucose or sugar*))))  OR (TITLE-ABS-KEY((lipid* W/1 (blood or level or profile*))))  OR (TITLE-ABS-KEY(("blood pressure" or "cardiometabolic syndrome*" or "C-peptide" or "C peptide" or cholesterol or HDL or LDL or VLDL or "connecting peptide" or "diastolic pressure" or "dysmetabolic syndrome*" or "glucose intolerance*" or "HOMA-IR" or "HOMA IR" or hyperglycemia* or hypertension or "insulin resistance" or "insulin sensitivity" or "metabolic syndrome*" or "metabolic cardiovascular syndrome*" or "pulse pressure" or "reaven syndrome X" or "systolic pressure")))  OR (TITLE-ABS-KEY(((glycated or glycosylated) W/1 (haemoglobin* or hemoglobin*))))  OR (TITLE-ABS-KEY(("glycohemoglobin A" or "Hb A1" or HbA1 or "Hb A1a-1" or "Hb A1a-2" or "Hb A1a b" or "Hb A1b" or "Hb A1c" or HbA1c or "hemoglobin A(1)" or "hemoglobin A1C")))  OR (TITLE-ABS-KEY(("Apo-B" or "Apo B" or ApoA or "ApoA-II" or "ApoA II" or "Apo A-V" or "Apo A V" or "Apo A1" or "Apo A2" or "Apo A5" or APOA5 or "Apo AI" or ApoB or ApoB48 or ApoC or "Apo C" or ApoC2 or "Apo D" or ApoD or ApoE or "Apo E" or "APOE-epsilon*" or "APOE epsilon*" or ApoE2 or "Apo E2" or "Apo E3" or ApoE3 or "Apo E4" or ApoE4 or ApoL or ApoL1 or apolipoprotein* or apoprotein* or enzactin or "glycerol trioleate" or proapolipoprotein* or triacetin or "triacetyl-glycerol*" or "triacetyl glycerol*" or triacetylglycerol* or triacylglycerol* or trielaidin or triglyceride* or "trioleate-glycerin" or "trioleate glycerin" or triolein or trioleoylglycerol or "trioleyl glycerol*"))))  AND ((TITLE-ABS-KEY(((bean* or chickpea* or fruit* or grain* or legume* or lentil* or nut or nuts or plant* or pea or peas or pulse* or seed* or soy* or vegetable*) W/3 protein*))) OR ((TITLE-ABS-KEY(((consum* or diet* or eat or eating or fed or feed or food* or ingest* or intake* or nutrition or nutrient* or source*) W/2 protein*))) AND (TITLE-ABS-KEY((bean* or chickpea* or fruit* or grain* or legume* or lentil* or nut or nuts or plant* or pea or peas or pulse* or seed* or soy* or vegetable*)))))  AND ( EXCLUDE ( DOCTYPE,"re" ) OR EXCLUDE ( DOCTYPE,"cp" ) OR EXCLUDE ( DOCTYPE,"ch" ) OR EXCLUDE ( DOCTYPE,"ed" ) OR EXCLUDE ( DOCTYPE,"le" ) OR EXCLUDE ( DOCTYPE,"no" ) OR EXCLUDE ( DOCTYPE,"sh" ) OR EXCLUDE ( DOCTYPE,"bk" ) OR EXCLUDE ( DOCTYPE,"cr" ) ) | |
