# Supplementary material for: Animal versus plant-based protein and risk of cardiovascular disease and type 2 diabetes: a systematic review of randomized controlled trials and prospective cohort studies
Source: Food Nutr Res. 2023 Mar 28;67:10.29219/fnr.v67.9003. doi: 10.29219/fnr.v67.9003 (PMC10084508; doi:10.29219/fnr.v67.9003)
Supplement: Supplementary file 2 [file FNR-67-9003-s002.docx]

Table S1. Excluded articles with reasons for exclusion

|  | Reference | Reason for exclusion |
| --- | --- | --- |
| 1 | Akter S, Mizoue T, Nanri A, Goto A, Noda M, Sawada N, et al. Low carbohydrate diet and all cause and cause-specific mortality. Clinical Nutrition. 2020;23:23. | Wrong exposure |
| 2 | Allen JK, Becker DM, Kwiterovich PO, Lindenstruth KA, Curtis C. Effect of soy protein-containing isoflavones on lipoproteins in postmenopausal women. Menopause. 2007;14(1):106-14. | High or unknown isoflavone content |
| 3 | Alonso A, Beunza JJ, Bes-Rastrollo M, Pajares RM, Martinez-Gonzalez MA. Vegetable protein and fiber from cereal are inversely associated with the risk of hypertension in a Spanish cohort. Archives of Medical Research. 2006;37(6):778-86 | Wrong study design (parallel comparison of animal and plant protein, but no substitution) |
| 4 | Al-Shaar L, Satija A, Wang DD, Rimm EB, Smith-Warner SA, Stampfer MJ, et al. Red meat intake and risk of coronary heart disease among US men: prospective cohort study. Bmj. 2020;371:m4141 | Wrong exposure |
| 5 | Altorf-van der Kuil W, Engberink MF, Geleijnse JM, Boer JM, Monique Verschuren WM. Sources of dietary protein and risk of hypertension in a general Dutch population. British Journal of Nutrition. 2012;108(10):1897-903. | Wrong study design (parallel comparison of animal and plant protein, but no substitution) |
| 6 | Altorf-van der Kuil W, Engberink MF, van Rooij FJ, Hofman A, van't Veer P, Witteman JC, et al. Dietary protein and risk of hypertension in a Dutch older population: the Rotterdam study. Journal of Hypertension. 2010;28(12):2394-400. | Wrong study design (parallel comparison of animal and plant protein, but no substitution) |
| 7 | Appel LJ, Sacks FM, Carey VJ, Obarzanek E, Swain JF, Miller ER, 3rd, et al. Effects of protein, monounsaturated fat, and carbohydrate intake on blood pressure and serum lipids: results of the OmniHeart randomized trial. Jama. 2005;294(19):2455-64. | Wrong exposure |
| 8 | Ashton E, Ball M. Effects of soy as tofu vs meat on lipoprotein concentrations. European Journal of Clinical Nutrition. 2000;54(1):14-9. | High or unknown isoflavone content |
| 9 | Ashton EL, Dalais FS, Ball MJ. Effect of meat replacement by tofu on CHD risk factors including copper induced LDL oxidation. Journal of the American College of Nutrition. 2000;19(6):761-7 | High or unknown isoflavone content |
| 10 | Azadbakht L, Kimiagar M, Mehrabi Y, Esmaillzadeh A, Padyab M, Hu FB, et al. Soy inclusion in the diet improves features of the metabolic syndrome: a randomized crossover study in postmenopausal women. American Journal of Clinical Nutrition. 2007;85(3):735-41 | High or unknown isoflavone content |
| 11 | Azadbakht L, Shakerhosseini R, Atabak S, Jamshidian M, Mehrabi Y, Esmaill-Zadeh A. Beneficiary effect of dietary soy protein on lowering plasma levels of lipid and improving kidney function in type II diabetes with nephropathy. European Journal of Clinical Nutrition. 2003;57(10):1292-4. | Wrong population |
| 12 | Baik I, Lee M, Jun NR, Lee JY, Shin C. A healthy dietary pattern consisting of a variety of food choices is inversely associated with the development of metabolic syndrome. Nutrition Research & Practice. 2013;7(3):233-41. | Wrong exposure |
| 13 | Bakhit RM, Klein BP, Essex-Sorlie D, Ham JO, Erdman JW, Jr., Potter SM. Intake of 25 g of soybean protein with or without soybean fiber alters plasma lipids in men with elevated cholesterol concentrations. Journal of Nutrition. 1994;124(2):213-22. | High or unknown isoflavone content |
| 14 | Bakhtiari A, Hajian-Tilaki K, Omidvar S, Nasiri-Amiri F. Clinical and metabolic response to soy administration in older women with metabolic syndrome: a randomized controlled trial. Diabetology & metabolic syndrome. 2019;11:47. | Wrong exposure |
| 15 | Bakhtiary A, Yassin Z, Hanachi P, Rahmat A, Ahmad Z, Halalkhor S, et al. Evaluation of the oxidative stress and glycemic control status in response to soy in older women with the metabolic syndrome. Iranian Red Crescent Medical Journal. 2011;13(11):795-804. | Wrong exposure |
| 16 | Bakhtiary A, Yassin Z, Hanachi P, Rahmat A, Ahmad Z, Jalali F. Effects of soy on metabolic biomarkers of cardiovascular disease in elderly women with metabolic syndrome. Archives of Iranian Medicine. 2012;15(8):462-8. | Wrong exposure |
| 17 | Baum J, Erdman Jr JW, Klein BP, Freels S, Perskvf V, Bakfrit RM, et al. Long term effects of soy protein and isoflavones on plasma lipid profiles in postmenopausal women. FASEB Journal. 1996;10(3). | High or unknown isoflavone content |
| 18 | Baum JA, Teng H, Erdman JW, Jr., Weigel RM, Klein BP, Persky VW, et al. Long-term intake of soy protein improves blood lipid profiles and increases mononuclear cell low-density-lipoprotein receptor messenger RNA in hypercholesterolemic, postmenopausal women. American Journal of Clinical Nutrition. 1998;68(3):545-51 | High or unknown isoflavone content |
| 19 | Bazzano LA, He J, Ogden LG, Loria C, Vupputuri S, Myers L, et al. Legume consumption and risk of coronary heart disease in US men and women: NHANES I Epidemiologic Follow-up Study. Archives of Internal Medicine. 2001;161(21):2573-8. | Wrong exposure |
| 20 | Becerra-Tomas N, Babio N, Martinez-Gonzalez MA, Corella D, Estruch R, Ros E, et al. Replacing red meat and processed red meat for white meat, fish, legumes or eggs is associated with lower risk of incidence of metabolic syndrome. Clinical Nutrition. 2016;35(6):1442-9. | Wrong exposure |
| 21 | Becerra-Tomas N, Diaz-Lopez A, Rosique-Esteban N, Ros E, Buil-Cosiales P, Corella D, et al. Legume consumption is inversely associated with type 2 diabetes incidence in adults: A prospective assessment from the PREDIMED study. Clinical Nutrition. 2018;37(3):906-13. | Wrong exposure |
| 22 | Belin RJ, Greenland P, Allison M, Martin L, Shikany JM, Larson J, et al. Diet quality and the risk of cardiovascular disease: The Women's Health Initiative (WHI). American Journal of Clinical Nutrition. 2011;94(1):49-57. | Wrong exposure |
| 23 | Bergeron N, Chiu S, Williams PT, S MK, Krauss RM. Effects of red meat, white meat, and nonmeat protein sources on atherogenic lipoprotein measures in the context of low compared with high saturated fat intake: a randomized controlled trial. 2019;110(1):24-33. | Wrong exposure |
| 24 | Bernstein AM, Pan A, Rexrode KM, Stampfer M, Hu FB, Mozaffarian D, et al. Dietary protein sources and the risk of stroke in men and women. Stroke. 2012;43(3):637-44. | Wrong exposure |
| 25 | Bernstein AM, Sun Q, Hu FB, Stampfer MJ, Manson JE, Willett WC. Major dietary protein sources and risk of coronary heart disease in women. Circulation. 2010;122(9):876-83. | Wrong exposure |
| 26 | Blum A, Lang N, Vigder F, Israeli P, Gumanovsky M, Lupovitz S, et al. Effects of soy protein on endothelium-dependent vasodilatation and lipid profile in postmenopausal women with mild hypercholesterolemia. Clinical & Investigative Medicine - Medecine Clinique et Experimentale. 2003;26(1):20-6. | High or unknown isoflavone content |
| 27 | Borodin EA, Menshikova IG, Dorovskikh VA, Feoktistova NA, Shtarberg MA, Yamamoto T, et al. Effects of two-month consumption of 30 g a day of soy protein isolate or skimmed curd protein on blood lipid concentration in Russian adults with hyperlipidemia. Journal of Nutritional Science & Vitaminology. 2009;55(6):492-7. | High or unknown isoflavone content |
| 28 | Brand vd, t PA. Red meat, processed meat, and other dietary protein sources and risk of overall and cause-specific mortality in The Netherlands Cohort Study. European Journal of Epidemiology.34(4):351-69. | Wrong exposure |
| 29 | Brussaard JH, van Raaij JM, Stasse-Wolthuis M, Katan MB, Hautvast JG. Blood pressure and diet in normotensive volunteers: absence of an effect of dietary fiber, protein, or fat. American Journal of Clinical Nutrition. 1981;34(10):2023-9. | High or unknown isoflavone content |
| 30 | Buendia JR, Bradlee ML, Singer MR, Moore LL. Diets higher in protein predict lower high blood pressure risk in Framingham Offspring Study adults. American Journal of Hypertension. 2015;28(3):372-9. | Wrong study design (parallel comparison of animal and plant protein, but no substitution) |
| 31 | Burke V, Hodgson JM, Beilin LJ, Giangiulioi N, Rogers P, Puddey IB. Dietary protein and soluble fiber reduce ambulatory blood pressure in treated hypertensives. Hypertension. 2001;38(4):821-6. | Wrong exposure |
| 32 | Campbell SC, Khalil DA, Payton ME, Arjm, i BH. One-year soy protein supplementation does not improve lipid profile in postmenopausal women. Menopause.17(3):587-93. | High or unknown isoflavone content |
| 33 | Chan R, Leung J, Woo J. High Protein Intake Is Associated with Lower Risk of All-Cause Mortality in Community-Dwelling Chinese Older Men and Women. Journal of Nutrition, Health & Aging. 2019;23(10):987-96. | Wrong study design (parallel comparison of animal and plant protein, but no substitution) |
| 34 | Chen Y, McClintock TR, Segers S, Parvez F, Islam T, Ahmed A, et al. Prospective investigation of major dietary patterns and risk of cardiovascular mortality in Bangladesh. International Journal of Cardiology.167(4):1495-501. | Wrong exposure |
| 35 | Chen Z, Franco OH, Lamballais S, Ikram MA, Schoufour JD, Muka T, et al. Associations of specific dietary protein with longitudinal insulin resistance, prediabetes and type 2 diabetes: The Rotterdam Study. Clinical Nutrition.39(1):242-9. | Wrong study design (parallel comparison of animal and plant protein, but no substitution)  (Retrieved from a systematic review) |
| 36 | Christie DR, Grant J, Darnell BE, Chapman VR, Gastaldelli A, Sites CK. Metabolic effects of soy supplementation in postmenopausal Caucasian and African American women: a randomized, placebo-controlled trial. American Journal of Obstetrics & Gynecology.203(2):153.e1-9. | High or unknown isoflavone content |
| 37 | Dai M, Tian L, Zi W, Cai B, Xiao L, Liu K, et al. Premorbid dietary intake of protein is associated with early outcomes but not with severity of ischemic stroke. Asia Pacific Journal of Clinical Nutrition. 2018;27(1):246-52. | Wrong study design (parallel comparison of animal and plant protein, but no substitution) |
| 38 | Dalais FS, Ebeling PR, Kotsopoulos D, McGrath BP, Teede HJ. The effects of soy protein containing isoflavones on lipids and indices of bone resorption in postmenopausal women. Clinical Endocrinology.58(6):704-9. | High or unknown isoflavone content |
| 39 | Das A , R. Cumming, V. Naganathan, F. Blyth, D. G. L. Couteur, D. J. Handelsman, et al.  Associations between dietary intake of total protein and sources of protein (plant vs. animal) and risk of all-cause and cause-specific mortality in older Australian men: The Concord Health and Ageing in Men Project ournal of Human Nutrition & Dietetics 2021 Vol. 21 21 | Wrong exposure(Not substitution) (parallel comparison of animal and plant protein, but no substitution) |
| 40 | de Koning L, Fung TT, Liao X, Chiuve SE, Rimm EB, Willett WC, et al. Low-carbohydrate diet scores and risk of type 2 diabetes in men. American Journal of Clinical Nutrition.93(4):844-50. | Wrong exposure |
| 41 | Desroches S, Mauger JF, Ausman LM, Lichtenstein AH, Lamarche B. Soy protein favorably affects LDL size independently of isoflavones in hypercholesterolemic men and women. J Nutr. 2004 Mar;134(3):574-9 | Same cohort and results as an included study ( Lichtenstein et al) |
| 42 | Evans M, Njike VY, Hoxley M, Pearson M, Katz DL. Effect of soy isoflavone protein and soy lecithin on endothelial function in healthy postmenopausal women. Menopause.14(1):141-9. | High or unknown isoflavone content |
| 43 | Farvid MS, Malekshah AF, Pourshams A, Poustchi H, Sepanlou SG, Sharafkhah M, et al. Dietary Protein Sources and All-Cause and Cause-Specific Mortality: The Golestan Cohort Study in Iran. American Journal of Preventive Medicine.52(2):237-48. | Wrong exposure |
| 44 | George KS, Munoz J, Akhavan NS, Foley EM, Siebert SC, Tenenbaum G, et al. Is soy protein effective in reducing cholesterol and improving bone health? Food & Function.11(1):544-51. | High or unknown isoflavone content |
| 45 | Gonciulea AR, Sellmeyer DE. The effect of dietary protein source on serum lipids: Secondary data analysis from a randomized clinical trial. Journal of Clinical Lipidology.11(1):46-54. | High or unknown isoflavone content |
| 46 | Grundy SM, Abrams JJ. Comparison of actions of soy protein and casein on metabolism of plasma lipoproteins and cholesterol in humans. American Journal of Clinical Nutrition.38(2):245-52. | High or unknown isoflavone content |
| 47 | Halton TL, Liu S, Manson JE, Hu FB. Low-carbohydrate-diet score and risk of type 2 diabetes in women. American Journal of Clinical Nutrition.87(2):339-46. | Wrong exposure |
| 48 | Halton TL, Willett WC, Liu S, Manson JE, Albert CM, Rexrode K, et al. Low-carbohydrate-diet score and the risk of coronary heart disease in women. New England Journal of Medicine.355(19):1991-2002. | Wrong exposure |
| 49 | Haring B, Gronroos N, Nettleton JA, von Ballmoos MC, Selvin E, Alonso A. Dietary protein intake and coronary heart disease in a large community based cohort: results from the Atherosclerosis Risk in Communities (ARIC) study corrected. PLoS ONE [Electronic Resource]. 2014;9(10):e109552. | Wrong study design (parallel comparison of animal and plant protein, but no substitution) |
| 50 | Haring B, Misialek JR, Rebholz CM, Petruski-Ivleva N, Gottesman RF, Mosley TH, et al. Association of Dietary Protein Consumption With Incident Silent Cerebral Infarcts and Stroke: The Atherosclerosis Risk in Communities (ARIC) Study. Stroke.46(12):3443-50. | Wrong study design (parallel comparison of animal and plant protein, but no substitution) |
| 51 | Haub MD, Wells AM, Campbell WW. Beef and soy-based food supplements differentially affect serum lipoprotein-lipid profiles because of changes in carbohydrate intake and novel nutrient intake ratios in older men who resistive-train. Metabolism: Clinical & Experimental.54(6):769-74. | High or unknown isoflavone content |
| 52 | He J, Wofford MR, Reynolds K, Chen J, Chen CS, Myers L, et al. Effect of dietary protein supplementation on blood pressure: a randomized, controlled trial. Circulation.124(5):589-95. | High or unknown isoflavone content |
| 53 | Hou W , J. Gao, W. Jiang, W. Wei, H. Wu, Y. Zhang, et al. Meal Timing of Subtypes of Macronutrients Consumption With Cardiovascular Diseases: NHANES, 2003 to 2016  Journal of Clinical Endocrinology & Metabolism 2021 Vol. 106(7 ) e2480-e2490 | Wrong exposure (Only intake at dinner  )  (parallel comparison of animal and plant protein, but no substitution) |
| 54 | Hu FB, Stampfer MJ, Manson JE, Rimm E, Colditz GA, Speizer FE, et al. Dietary protein and risk of ischemic heart disease in women. American Journal of Clinical Nutrition.70(2):221-7. | Wrong study design (parallel comparison of animal and plant protein, but no substitution) |
| 55 | Kelemen LE, Kushi LH, Jacobs DR, Jr., Cerhan JR. Associations of dietary protein with disease and mortality in a prospective study of postmenopausal women. American Journal of Epidemiology.161(3):239-49. | Wrong study design (parallel comparison of animal and plant protein, but no substitution) |
| 56 | Kreijkamp-Kaspers S, Kok L, Bots ML, Grobbee DE, Lampe JW, van der Schouw YT. Randomized controlled trial of the effects of soy protein containing isoflavones on vascular function in postmenopausal women. American Journal of Clinical Nutrition.81(1):189-95. | High or unknown isoflavone content |
| 57 | Kreijkamp-Kaspers S, Kok L, Grobbee DE, de Haan EH, Aleman A, Lampe JW, et al. Effect of soy protein containing isoflavones on cognitive function, bone mineral density, and plasma lipids in postmenopausal women: a randomized controlled trial. Jama.292(1):65-74. | High or unknown isoflavone content |
| 58 | Langsetmo L, Harrison S, Jonnalagadda S, Pereira SL, Shikany JM, Farsijani S, et al. Low Protein Intake Irrespective of Source is Associated with Higher Mortality Among Older Community-dwelling Men. Journal of Nutrition, Health & Aging. 2020;24(8):900-5. | Wrong study design (parallel comparison of animal and plant protein, but no substitution) |
| 59 | Larsson SC, Virtamo J, Wolk A. Dietary protein intake and risk of stroke in women. Atherosclerosis.224(1):247-51. | Wrong study design (parallel comparison of animal and plant protein, but no substitution) |
| 60 | Lelong H, Blacher J, Baudry J, Adriouch S, Galan P, Fezeu L, et al. Individual and Combined Effects of Dietary Factors on Risk of Incident Hypertension: Prospective Analysis From the NutriNet-Sante Cohort. Hypertension.70(4):712-20. | Wrong study design (parallel comparison of animal and plant protein, but no substitution) |
| 61 | Ma Y, Chiriboga D, Olendzki BC, Nicolosi R, Merriam PA, Ockene IS. Effect of soy protein containing isoflavones on blood lipids in moderately hypercholesterolemic adults: a randomized controlled trial. Journal of the American College of Nutrition.24(4):275-85. | High or unknown isoflavone content |
| 62 | Maki KC, Butteiger DN, Rains TM, Lawless A, Reeves MS, Schasteen C, et al. Effects of soy protein on lipoprotein lipids and fecal bile acid excretion in men and women with moderate hypercholesterolemia. Journal of Clinical Lipidology.4(6):531-42. | High or unknown isoflavone content |
| 63 | Matthan NR, Jalbert SM, Ausman LM, Kuvin JT, Karas RH, Lichtenstein AH. Effect of soy protein from differently processed products on cardiovascular disease risk factors and vascular endothelial function in hypercholesterolemic subjects. American Journal of Clinical Nutrition.85(4):960-6. | High or unknown isoflavone content |
| 64 | Merono T , R. Zamora-Ros, N. Hidalgo-Liberona, M. Rabassa, S. Bandinelli, L. Ferrucci, et al.  Animal protein intake is inversely associated with mortality in older adults: the InCHIANTI study  Journals of Gerontology Series A Biological Sciences & Medical Sciences 2021 Vol. 27 , 27 | Wrong exposure(Not substitution) (parallel comparison of animal and plant protein, but no substitution) |
| 65 | Mitchell JH, Collins AR. Effects of a soy milk supplement on plasma cholesterol levels and oxidative DNA damage in men--a pilot study. European Journal of Nutrition.38(3):143-8. | High or unknown isoflavone content |
| 66 | Nilausen K, Meinertz H. Variable lipemic response to dietary soy protein in healthy, normolipemic men. American Journal of Clinical Nutrition.68(6):1380S-4S. | High or unknown isoflavone content |
| 67 | Nilausen K, Meinertz H. Lipoprotein(a) and dietary proteins: casein lowers lipoprotein(a) concentrations as compared with soy protein. American Journal of Clinical Nutrition.69(3):419-25. | High or unknown isoflavone content |
| 68 | Ozawa M, Yoshida D, Hata J, Ohara T, Mukai N, Shibata M, et al. Dietary Protein Intake and Stroke Risk in a General Japanese Population: The Hisayama Study. Stroke.48(6):1478-86. | Wrong study design (parallel comparison of animal and plant protein, but no substitution) |
| 69 | Preis SR, Stampfer MJ, Spiegelman D, Willett WC, Rimm EB. Dietary protein and risk of ischemic heart disease in middle-aged men. American Journal of Clinical Nutrition.92(5):1265-72. | Wrong study design (parallel comparison of animal and plant protein, but no substitution) |
| 70 | Preis SR, Stampfer MJ, Spiegelman D, Willett WC, Rimm EB. Lack of association between dietary protein intake and risk of stroke among middle-aged men. American Journal of Clinical Nutrition.91(1):39-45. | Wrong study design (parallel comparison of animal and plant protein, but no substitution) |
| 71 | Prentice R. L., M. Pettinger, C. Zheng, M. L. Neuhouser, D. Raftery, G. A. N. Gowda, et al.  Biomarkers for Components of Dietary Protein and Carbohydrate with Application to Chronic Disease Risk in Postmenopausal Women  Journal of Nutrition 2022 Vol. 152 Issue 4 1107-1117 | No plant protein. Wrong exposure(Not substitution (parallel comparison of animal and plant protein, but no substitution) |
| 72 | Prescott SL, Jenner DA, Beilin LJ, Margetts BM, ongen R. Controlled study of the effects of dietary protein on blood pressure in normotensive humans. Clinical & Experimental Pharmacology & Physiology.14(3):159-62. | High or unknown isoflavone content |
| 73 | Prescott SL, Jenner DA, Beilin LJ, Margetts BM, ongen R. A randomized controlled trial of the effect on blood pressure of dietary non-meat protein versus meat protein in normotensive omnivores. Clinical Science.74(6):665-72. | High or unknown isoflavone content |
| 74 | Razavi A. C., L. A. Bazzano, J. He, S. P. Whelton, C. Fernandez, S. Ley, et al.  Consumption of animal and plant foods and risk of left ventricular diastolic dysfunction: the Bogalusa Heart Study  ESC Heart Failure 2020 Vol. 7 Issue 5 2700-2710 | Wrong exposure, No substitution analysis of protein, only food groups |
| 75 | Roughead ZK, Hunt JR, Johnson LK, Badger TM, Lykken GI. Controlled substitution of soy protein for meat protein: effects on calcium retention, bone, and cardiovascular health indices in postmenopausal women. Journal of Clinical Endocrinology & Metabolism.90(1):181-9 | High or unknown isoflavone content |
| 76 | Ruscica M, Pavanello C, ini S, Gomaraschi M, Vitali C, Macchi C, et al. Effect of soy on metabolic syndrome and cardiovascular risk factors: a randomized controlled trial. European Journal of Nutrition.57(2):499-511. | High or unknown isoflavone content |
| 77 | Shang X, Scott D, Hodge A, English DR, Giles GG, Ebeling PR, Sanders KM. Dietary protein from different food sources, incident metabolic syndrome and changes in its components: An 11-year longitudinal study in healthy community-dwelling adults. Clin Nutr. 2017 Dec;36(6):1540-1548. | Wrong exposure(Not substitution) (parallel comparison of animal and plant protein, but no substitution) |
| 78 | Shang X, Scott D, Hodge AM, English DR, Giles GG, Ebeling PR, Sanders KM. Dietary protein intake and risk of type 2 diabetes: results from the Melbourne Collaborative Cohort Study and a meta-analysis of prospective studies. Am J Clin Nutr. 2016 Nov;104(5):1352-1365 | Wrong exposure(Not substitution )(parallel comparison of animal and plant protein, but no substitution) |
| 79 | Shorey RL, Bazan B, Lo GS, Steinke FH. Determinants of hypocholesterolemic response to soy and animal protein-based diets. American Journal of Clinical Nutrition.34(9):1769-78. | High or unknown isoflavone content |
| 80 | Sites CK, Cooper BC, Toth MJ, Gastaldelli A, Arabshahi A, Barnes S. Effect of a daily supplement of soy protein on body composition and insulin secretion in postmenopausal women. Fertility & Sterility.88(6):1609-17. | High or unknown isoflavone content |
| 81 | Sluijs I, Beulens JW, van der A DL, Spijkerman AM, Grobbee DE, van der Schouw YT. Dietary intake of total, animal, and vegetable protein and risk of type 2 diabetes in the European Prospective Investigation into Cancer and Nutrition (EPIC)-NL study. Diabetes Care. 2010 Jan;33(1):43-8. | Wrong exposure(Not substitution) |
| 82 | Sluik D, Brouwer-Brolsma EM, Berendsen AAM, Mikkila V, Poppitt SD, Silvestre MP, et al. Protein intake and the incidence of pre-diabetes and diabetes in 4 population-based studies: the PREVIEW project. American Journal of Clinical Nutrition.109(5):1310-8. | Wrong study design (parallel comparison of animal and plant protein, but no substitution) |
| 83 | Stamler J, Liu K, Ruth KJ, Pryer J, Greenl, P. Eight-year blood pressure change in middle-aged men: relationship to multiple nutrients. Hypertension.39(5):1000-6. | Wrong outcome |
| 84 | Sugihiro T, Yoneda M, Ohno H, Oki K, Hattori N. Associations of nutrient intakes with obesity and diabetes mellitus in the longitudinal medical surveys of Japanese Americans. Journal of Diabetes Investigation.10(5):1229-36. | Wrong study design (parallel comparison of animal and plant protein, but no substitution) |
| 85 | Teede HJ, Dalais FS, Kotsopoulos D, Liang YL, Davis S, McGrath BP. Dietary soy has both beneficial and potentially adverse cardiovascular effects: a placebo-controlled study in men and postmenopausal women. Journal of Clinical Endocrinology & Metabolism.86(7):3053-60. | High or unknown isoflavone content |
| 86 | Teixeira SR, Potter SM, Weigel R, Hannum S, Erdman JW, Jr., Hasler CM. Effects of feeding 4 levels of soy protein for 3 and 6 wk on blood lipids and apolipoproteins in moderately hypercholesterolemic men. American Journal of Clinical Nutrition.71(5):1077-84. | High or unknown isoflavone content |
| 87 | Tharrey M, Mariotti F, Mashchak A, Barbillon P, Delattre M, Fraser GE. Patterns of plant and animal protein intake are strongly associated with cardiovascular mortality: the Adventist Health Study-2 cohort. International Journal of Epidemiology.47(5):1603-12. | Wrong study design (parallel comparison of animal and plant protein, but no substitution) |
| 88 | Thorp AA, Howe PR, Mori TA, Coates AM, Buckley JD, Hodgson J, et al. Soy food consumption does not lower LDL cholesterol in either equol or nonequol producers. American Journal of Clinical Nutrition.88(2):298-304. | High or unknown isoflavone content |
| 89 | Tielemans SM, Kromhout D, Altorf-van der Kuil W, Geleijnse JM. Associations of plant and animal protein intake with 5-year changes in blood pressure: the Zutphen Elderly Study. Nutrition Metabolism & Cardiovascular Diseases.24(11):1228-33. | Wrong outcome |
| 90 | Tonstad S, Smerud K, Hoie L. A comparison of the effects of 2 doses of soy protein or casein on serum lipids, serum lipoproteins, and plasma total homocysteine in hypercholesterolemic subjects. American Journal of Clinical Nutrition.76(1):78-84. | High or unknown isoflavone content |
| 91 | van Nielen M, Feskens EJ, Mensink M, Sluijs I, Molina E, Amiano P, et al. Dietary protein intake and incidence of type 2 diabetes in Europe: the EPIC-InterAct Case-Cohort Study. Diabetes Care.37(7):1854-62. | Wrong study design (parallel comparison of animal and plant protein, but no substitution) |
| 92 | van Nielen M, Feskens EJ, Rietman A, Siebelink E, Mensink M. Partly replacing meat protein with soy protein alters insulin resistance and blood lipids in postmenopausal women with abdominal obesity. Journal of Nutrition.144(9):1423-9. | High or unknown isoflavone content |
| 93 | van Raaij JM, Katan MB, Hautvast JG, Hermus RJ. Effects of casein versus soy protein diets on serum cholesterol and lipoproteins in young healthy volunteers. American Journal of Clinical Nutrition.34(7):1261-71. | High or unknown isoflavone content |
| 94 | van Raaij JM, Katan MB, West CE, Hautvast JG. Influence of diets containing casein, soy isolate, and soy concentrate on serum cholesterol and lipoproteins in middle-aged volunteers. American Journal of Clinical Nutrition.35(5):925-34. | High or unknown isoflavone content |
| 95 | Vigna GB, Pansini F, Bonaccorsi G, Albertazzi P, Donega P, Zanotti L, et al. Plasma lipoproteins in soy-treated postmenopausal women: a double-blind, placebo-controlled trial. Nutrition Metabolism & Cardiovascular Diseases.10(6):315-22. | High or unknown isoflavone content |
| 96 | Wang YF, Yancy WS, Jr., Yu D, Champagne C, Appel LJ, Lin PH. The relationship between dietary protein intake and blood pressure: results from the PREMIER study. Journal of Human Hypertension.22(11):745-54. | Wrong study design (parallel comparison of animal and plant protein, but no substitution) |
| 97 | West SG, Hilpert KF, Juturu V, Bordi PL, Lampe JW, Mousa SA, et al. Effects of including soy protein in a blood cholesterol-lowering diet on markers of cardiac risk in men and in postmenopausal women with and without hormone replacement therapy. Journal of Women's Health.14(3):253-62. | High or unknown isoflavone content |
| 98 | Wofford MR, Rebholz CM, Reynolds K, Chen J, Chen CS, Myers L, et al. Effect of soy and milk protein supplementation on serum lipid levels: a randomized controlled trial. European Journal of Clinical Nutrition.66(4):419-25. | High or unknown isoflavone content |
| 99 | Wolfe BM, Giovannetti PM. Elevation of VLDL-cholesterol during substitution of soy protein for animal protein in diets of hypercholesterolemic Canadians. Nutrition Reports International. 1985;32(5):1057-65. | High or unknown isoflavone content |
| 100 | Wolfe BM, Giovannetti PM, Cheng DCH. Hypolipidemic effect of substituting soybean protein isolate for all meat and dairy protein in the diets of hypercholesterolemic men. Nutrition Reports International. 1981;24(6):1187-98. | High or unknown isoflavone content |
| 101 | Wurtz AML, Jakobsen MU, Bertoia ML, Hou T, Schmidt EB, Willett WC, et al. Replacing the consumption of red meat with other major dietary protein sources and risk of type 2 diabetes mellitus: a prospective cohort study. American Journal of Clinical Nutrition.113(3):612-21. | Wrong exposure |
| 102 | Yildirir A, Tokgozoglu SL, Oduncu T, Oto A, Haznedaroglu I, Akinci D, et al. Soy protein diet significantly improves endothelial function and lipid parameters. Clinical Cardiology.24(11):711-6. | High or unknown isoflavone content |
| 103 | Zhong VW, Allen NB, Greenl, P, Carnethon MR, Ning H, et al. Protein foods from animal sources, incident cardiovascular disease and all-cause mortality: a substitution analysis. International Journal of Epidemiology.50(1):223-33. | Wrong exposure |
| 104 | Zhou C , C. Liu, Z. Zhang, M. Liu, Y. Zhang, H. Li, et al.  Variety and quantity of dietary protein intake from different sources and risk of new-onset diabetes: a Nationwide Cohort Study in China  BMC Medicine 2022 Vol. 20 Issue 1 Pages 6 | Wrong exposure(Not substitution )(parallel comparison of animal and plant protein, but no substitution) |
